# Oleoylethanolamide regulates intestinal stem cell activity and villus size via PPARα signaling pathway

**DOI:** 10.1002/imo2.70078

**Published:** 2026-01-26

**Authors:** Qianqian Wang, Lanmei Yin, Zhaobin Wang, Jun Li, Xianglin Zeng, Qiye Wang, Jianzhong Li, Kang Xu, Huansheng Yang, Yulong Yin

**Affiliations:** ^1^ Hunan Provincial Key Laboratory of Animal Intestinal Function and Regulation, Hunan International Joint Laboratory of Animal Intestinal Ecology and Health, Laboratory of Animal Nutrition and Human Health, College of Life Sciences Hunan Normal University Changsha China; ^2^ Key Laboratory of Agro‐ecological Processes in Subtropical Region, Institute of Subtropical Agriculture, Chinese Academy of Sciences; Hunan Provincial Engineering Research Center for Healthy Livestock and Poultry Production; Scientific Observing and Experimental Station of Animal Nutrition and Feed Science in South‐Central Ministry of Agriculture; Hunan Provincial Key Laboratory of Animal Nutritional Physiology and Metabolic Process Changsha China; ^3^ Department of Animal Science University of Manitoba Winnipeg Canada; ^4^ Yuelushan Laboratory Changsha China

**Keywords:** intestinal stem cells, intestinal villus size, lipid metabolism, oleoylethanolamide, nutrient digestibility

## Abstract

The size of intestinal villi plays a critical role in determining digestive and absorptive functions, directly affecting nutrient digestibility and feed efficiency. Therefore, elucidating the key mechanisms regulating villus development is of significant importance. This study compares intestinal development in Shaziling pigs and Yorkshire pigs, which exhibit distinct lipid metabolism characteristics, revealing significant enrichment of different fatty acid metabolic pathways and the peroxisome proliferator‐activated receptors (PPAR) signaling pathway. LC‐MS metabolomics analysis further explores the potential correlation between changes in fatty acid metabolism and PPARα expression, identifying glycerophospholipid metabolism and its key metabolite oleoylethanolamide (OEA) as critical factors. Pharmacological interventions and genetic mouse models manipulating PPARα signaling demonstrate that OEA regulates stem cell activity and intestinal villus size via PPARα, with this effect linked to energy metabolism driven by oxidative phosphorylation. Notably, dietary supplementation with OEA or palmitoylethanolamide—another endogenous PPARα ligand increases villus size and nutrient digestibility. These findings indicate that lipid metabolism contributes to the regulation of intestinal villus size and uncover a novel mechanism by which N‐acylethanolamines modulate intestinal morphological development through the PPARα‐oxidative phosphorylation axis. The results provide a theoretical basis for developing novel feed additives based on endogenous lipid signaling molecules.

## Highlights

- Species‐specific lipid metabolism fundamentally links fatty acid metabolic pathways to variations in intestinal villus development.
- Oleoylethanolamide regulates intestinal stem cell activity and villus size through the PPARα‐oxidative phosphorylation axis.
- Dietary N‐acylethanolamines enhance intestinal villus size and nutrient digestibility as potential feed additives.

## Graphical abstract

First use of a natural swine model with lipid metabolism to directly link lipid differences to a quantifiable intestinal villus height phenotype. Identified the phospholipid‐derived oleoylethanolamide, rather than canonical fatty acids, as the endogenous primary ligand that activates peroxisome proliferator‐activated receptors α (PPARα) to enlarge villi. Established and validated a PPARα‐oxidative phosphorylation–stem cell energy switch axis, offering a druggable metabolic node that couples nutrient status to morphogenesis.

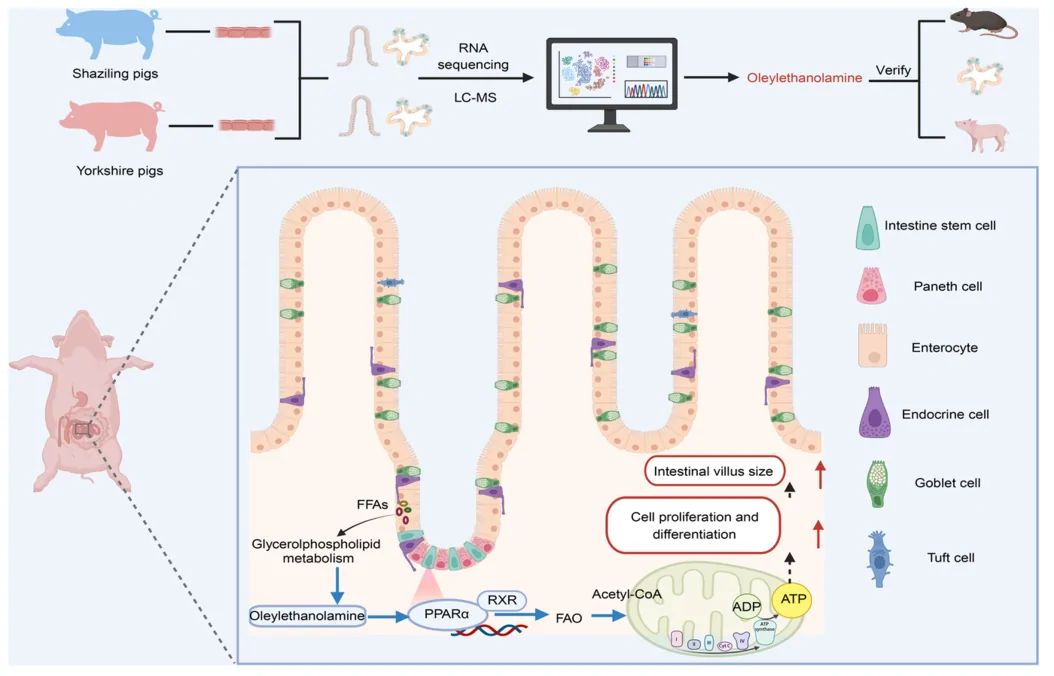

## 1 INTRODUCTION

Contemporary agricultural and food systems are under considerable pressure and face significant challenges. Despite global productivity gains, approximately 14 percent of the population still experiences food insecurity or chronic malnutrition [1]. Globally, roughly one‐third of arable land is allocated for animal feed production [2]. Feed‐related expenses constitute approximately 60%–70% of the total costs in pig production systems [3]. Consequently, enhancing feed efficiency in pigs is of paramount importance. The primary function of the intestine is to digest and absorb nutrients, which underpins the growth and development of pigs [4]. It has been reported that, from an animal breeding standpoint, the digestibility coefficient correlates positively with feed efficiency [5]. Increasing particle uniformity and reducing particle size enhance the contact surface area, thereby improving feed efficiency and nutrient digestibility [6]. The digestive capacity of the small intestine is also intimately linked to feed efficiency. Research indicates that nutrient digestibility is positively correlated with villus height (VH) and enterocyte proliferation in the small intestine [7]. Therefore, elucidating the key metabolic pathways regulating villus development is of significant importance for improving feed efficiency in pigs.

Morphological adaptations at the tissue and cellular levels place the intestinal epithelium in close and prolonged contact with the luminal contents, facilitating efficient nutrient absorption [8]. Morphological adaptations required for this absorbent surface include elongation of the intestinal tract, and production of villi [9]. The small intestinal mucosa forms many finger‐like folds of tissue called villi that project into the lumen, increasing the surface area available for nutrient absorption [10]. Complementary to these villi, crypts—invaginated structures at the epithelial base—harbor abundant stem cells, which give rise to progenitor cells that rapidly proliferate and eventually migrate up the villus and differentiate into absorptive enterocytes and secretory cells (endocrine cells and goblet cells), or differentiate into Paneth cells that migrate towards the base of the crypt [11]. Intestinal stem cell (ISC) homeostasis is pivotal for villus morphology, as it directly determines villus architecture by governing the continuous renewal and turnover of the intestinal epithelium. In recent years, lipid metabolism has been recognized as playing a crucial role in ISC proliferation, differentiation, and tissue homeostasis [12]. Existing studies have revealed that various lipid molecules and related pathways are implicated in the regulation of stem cell fate. Lipid signaling molecules, such as cholesterol [13], phosphatidic acid [14], and arachidonic acid [15], as well as critical pathways interacting with lipid metabolism, have all been reported to modulate ISC function. However, how changes in lipid metabolism of ISC affect their digestive and absorptive efficiency have not been specifically elucidated.

To better understand how lipid metabolism regulates stem cell function and villus development, we selected Shaziling pigs (an obese‐type breed) and Yorkshire pigs (a lean‐type breed) as comparative models to investigate the influence of differential lipid metabolism on intestinal development. Based on preliminary observations of distinct metabolic patterns between these models, we hypothesized that different lipid metabolic pathways might significantly impact intestinal morphology through specific signaling mechanisms. This study employs in vivo intestinal tissues and in vitro organoid models to identify the key lipid metabolic pathways and signaling receptors involved in regulating intestinal development, and determine how metabolic factors influence stem cell activity and villus morphogenesis through their downstream mechanisms. We further aimed to establish whether endogenous lipid signaling molecules directly connect with intestinal morphological development, thereby elucidating the impact of lipid metabolism on villus development.

## 2 RESULTS

### Shaziling pigs exhibit larger villus size but shorter small intestine

We initially selected six Shaziling pigs and six Yorkshire pigs at each of the following developmental stages: 30, 60, 90, 150, 210, and 300 days of age, to study differences in their intestinal development (Figure S1A). Shaziling pigs exhibited a significant increase in small intestine length and weight (Figure S1B,D), whereas the small intestine index (weight‐to‐body and length‐to‐body ratios) declined markedly with advancing age (Figure S1C,E). At the same time, there was an interaction effect between breed and age on their organ index.

Intestinal function, including digestive enzyme secretion and nutrient absorption, is closely linked to villus and crypt morphology. To assess this relationship, we analyzed intestinal morphology across developmental stages. Shaziling and Yorkshire pigs displayed distinct age‐dependent differences in intestinal architecture. VH in the duodenum, jejunum, and ileum peaked at 210 days of age in both breeds (Figure S1F–H), with Shaziling pigs showing greater VH and shallower crypt depth (CD) compared to Yorkshire pigs (Figure S1I–K). Consequently, Shaziling pigs exhibited a trend toward increased villus width (VW) (Figure S1L–N) and a higher villus height to crypt depth (VH/CD) ratio (Figure S1O–Q). Age‐associated increases in VH and surface area (Figure S1R–T) suggest progressive expansion of the intestinal absorptive capacity. Notably, breed‐specific variations in these morphological changes were modulated by age.

### Shaziling pigs exhibit enhanced ISC activity and nutrient transporter expression but similar nutrient digestibility compared to Yorkshire Pigs

The regulation of ISC proliferation and differentiation represents a primary mechanism for optimizing intestinal epithelial villus morphology. We quantified Ki67‐positive cells, endocrine cells, and goblet cells in jejunal tissues, with representative images provided in Figure S2. In Shaziling pigs, Ki67‐positive cell counts within jejunal crypts declined significantly with age progression, yet remained markedly higher than those of Yorkshire pigs (Figure S3A). Concurrently, Shaziling pigs exhibited age‐dependent increases in endocrine cells (Figure S3B,C) and goblet cells (Figure S3D,E) within both villi and crypts. Goblet cell counts in Shaziling jejunal villi (Figure S3D) were significantly elevated compared to Yorkshire pigs at 60, 90, and 150 days of age. While endocrine cell counts did not differ significantly between breeds, a significant breed × age interaction was observed.

VH is closely associated with small intestinal digestion and absorption capacity. We further assessed digestive enzyme activity and nutrient transporter mRNA expression. Alkaline phosphatase and disaccharidase activities in Shaziling pigs increased with age, peaking at 90 days, and were significantly higher than in Yorkshire pigs (Figure S3F–H). A significant breed × age interaction was identified for alkaline phosphatase and maltase. As the age increased to 90 days, relative mRNA expression levels of nutrient transporters in Shaziling jejunal mucosa progressively increased, surpassing Yorkshire pigs. This was accompanied by significant age‐dependent differences in the expression of *SGLT1* (Figure S3I), *SLC1A1* (Figure S3M), *PepT1* (Figure S3N), and *SLC27A4* (Figure S3O), and further breed‐specific differences in the expression of *SGLT1* (Figure S3I), *GLUT2* (Figure S3J), *SLC6A19* (Figure S3K), *SLC7A1* (Figure S3L), *SLC1A1* (Figure S3M), and *SLC27A4* (Figure S3O). However, whole‐gut gross energy and crude protein digestibility did not differ between breeds (Figure S3P,Q). These findings suggest divergent adaptive mechanisms for intestinal nutrient absorption: increased VH, elevated digestive enzyme activity, and enhanced nutrient transporter expression in Shaziling pigs may compensate for reduced small intestine length, thereby maintaining nutrient digestibility.

### Significant differences in lipid metabolism pathways between Shaziling and Yorkshire pigs

To investigate divergent intestinal development patterns between the two breeds, we conducted transcriptomic sequencing. In the KEGG enrichment analysis of intestinal tissues (Figure 1A), Shaziling pigs exhibited significant enrichment in nutrient absorption and lipid metabolism pathways compared to Yorkshire pigs, including fat digestion and absorption, cholesterol metabolism, arachidonic acid metabolism, steroid biosynthesis, glycerophospholipid metabolism, glycerolipid metabolism, and sphingolipid metabolism. These pathways were further corroborated by positive enrichment scores in the Gene Set Enrichment Analysis (GSEA) analysis (Figure 1B–H). To validate the transcriptome findings, we performed quantitative (Reverse Transcription‐Polymerase Chain Reaction) RT‐PCR, which revealed consistent mRNA expression patterns. Specifically, Shaziling pigs displayed elevated expression levels of fat digestion and absorption‐associated genes (*CD36*, *DGAT2*; Figure 1I), cholesterol metabolism‐associated genes (*CD36*, Figure 1J), arachidonic acid metabolism‐associated genes (*GGT1*, *GGT5*, *CYP2B22*; Figure 1K), steroid biosynthesis‐associated gene (*AGPAT4*; Figure 1L), Glycerophospholipid metabolism‐associated gene (*AGPAT4*, *PNPLA6*, Figure 1M), Glycerolipid metabolism‐associated gene (*DGAT2*, *ATPAT4*, Figure 1N), Sphingolipid metabolism‐associated gene (*SMPD1*, Figure 1O) compared to Yorkshire pigs.

**FIGURE 1:**
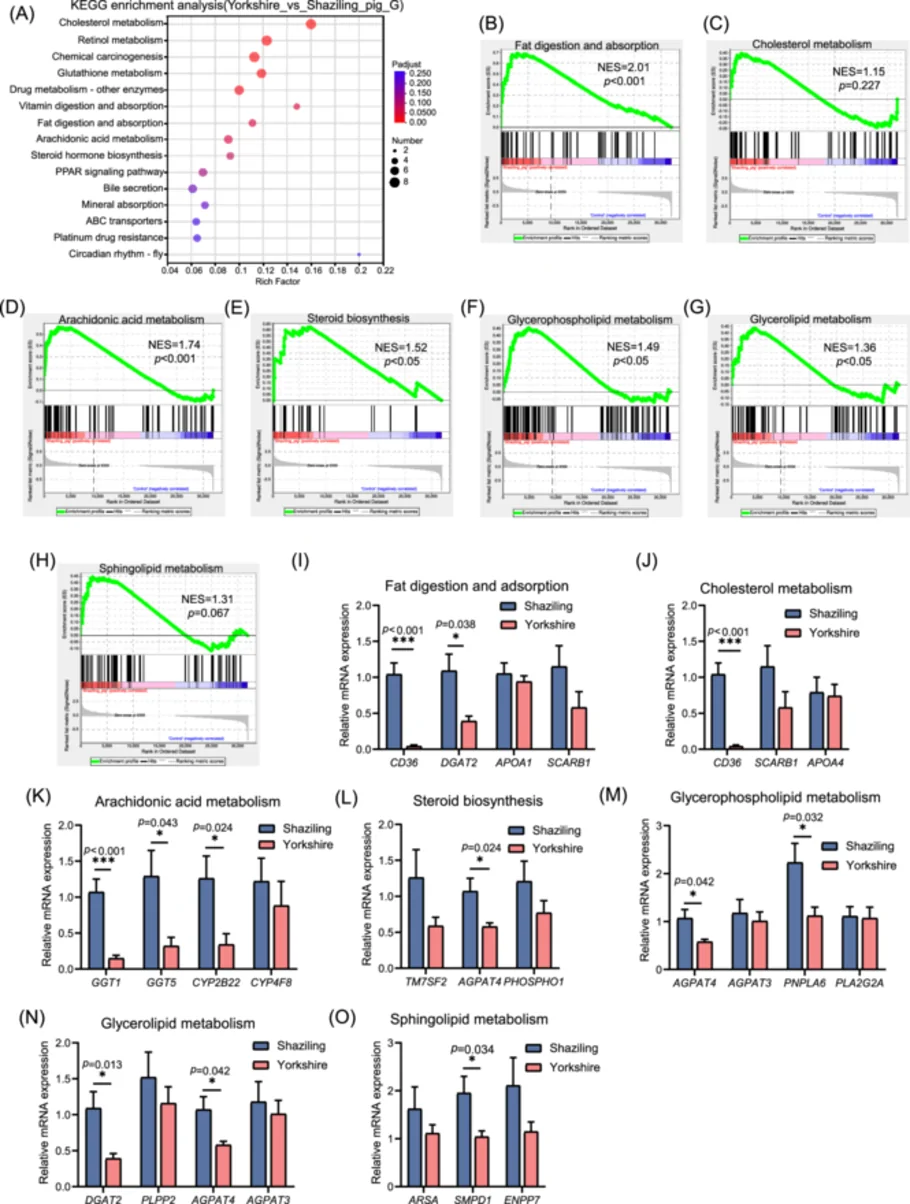
Significant differences in intestinal nutrient absorption and lipid metabolism pathways between Shaziling and Yorkshire pigs. (A) The KEGG pathway enrichment analysis between Shaziling and Yorkshire pigs at 210 days of age. GSEA enrichment maps for pathways related lipid metabolism (B) fat digestion and absorption, (C) cholesterol metabolism, (D) arachidonic acid metabolism, (E) steroid biosynthesis, (F) glycerophospholipid metabolism, (G) glycerolipid metabolism, (H) Sphingolipid metabolism. Relative expression of genes related to nutrient absorption and lipid metabolism pathways (I) fat digestion and absorption, (J) cholesterol metabolism, (K) arachidonic acid metabolism, (L) steroid biosynthesis, (M) Glycerophospholipid metabolism, (N) Glycerolipid metabolism, (O) Sphingolipid metabolism. KEGG, Kyoto Encyclopedia of Genes and Genomes; PPAR, peroxisome proliferator‐activated receptors; GSEA, Gene Set Enrichment Analysis; NES, Normalized Enrichment Score; *CD36*, cluster of differentination 36; *APOA1*, apolipoprotein A1; *DGAT2*, diacylglycerol O‐acyltransferase 2; *SCARB1*, scavenger receptor class B member 1; *APOA4*, apolipoprotein A4; *GGT1*, gamma‐glutamyltransferase 1; *GGT5*, gamma‐glutamyltransferase5; *CYP4F8*, cytochrome P450 family 4 subfamily F member 8; *CYP2B22*, cytochrome P450 family 2 subfamily B member 6B; *AGPAT3*, 1‐acylglycerol‐3‐phosphate O‐acyltransferase 3; *PNPLA6*, patatin‐like phospholipase domain containing 6; *PLA2G2A*, phospholipase A2, group IIA; *PLPP2*, Phospholipid Phosphatase 2; *ARSA*, Arylsulfatase A; *SMPD1*, Sphingomyelin Phosphodiesterase 1; *ENPP7,* ectonucleotide pyrophosphatase/phosphodiesterase 7; *TM7SF2*, transmembrane 7 superfamily member 2; *AGPAT4*, 1‐acylglycerol‐3‐phosphate O‐acyltransferase 4; *PHOSPHO1*, phosphocholine phosphatase 1. The results of fat metabolism gene expression between the two groups at 210 days of age were analyzed using an unpaired two‐tailed Student's *t*‐test. Data are presented as means ± SEM (*n* = 6 biological replicates/group). Significant differences between the two breeds at the same age are marked as* for *p* < 0.05, ** for *p* < 0.01, *** for *p* < 0.001.

### PPARα signaling pathway is more highly expressed in Shaziling pigs

Organoids, which mimic in vitro intestinal epithelial development, exhibit budding morphogenesis that closely recapitulates crypt‐villus axis dynamics during epithelial renewal [16]. Comparative analysis revealed that organoids derived from Shaziling pigs exhibited significantly greater budding length and growth area compared to those from Yorkshire pigs (Figure 2A,B). Similarly, immunohistochemical analysis by Olfactomedin 4+ (Olfm4+ ) showed that the number of stem cells in the intestinal crypts of Shaziling pigs was higher than that of Yorkshire pigs (Figure 2C,D). Transcriptomic profiling of organoids demonstrated significant enrichment of lipid metabolism pathways consistent with pathways identified in tissue samples (Figure 2E). Given that Peroxisome Proliferator‐Activated Receptor Alpha (PPARα) acts as a key transcription factor regulating fatty acid oxidation and related metabolic processes, we focused subsequent analyses on this pathway. PPARα is predominantly expressed in the proximal jejunal villus. Quantitative RT‐PCR confirmed upregulated PPARα pathway activity in Shaziling pigs (Figure 2F). To investigate the role of PPARα in intestinal growth, we employed an organoid culture system. Crypt‐derived organoids were treated with the selective PPARα antagonist GW6471 and agonist WY14643. GW6471 significantly inhibited organoid budding and growth, whereas WY14643 enhanced budding efficiency, budding length, and growth area (Figure 2G). Quantitative RT‐PCR confirmed elevated expression of PPARα target genes (Figure 2H) and ISC markers *Lgr5* and *Bmi1* (Figure 2I), the representative images of organoids were shown in Figure 2J.

**FIGURE 2:**
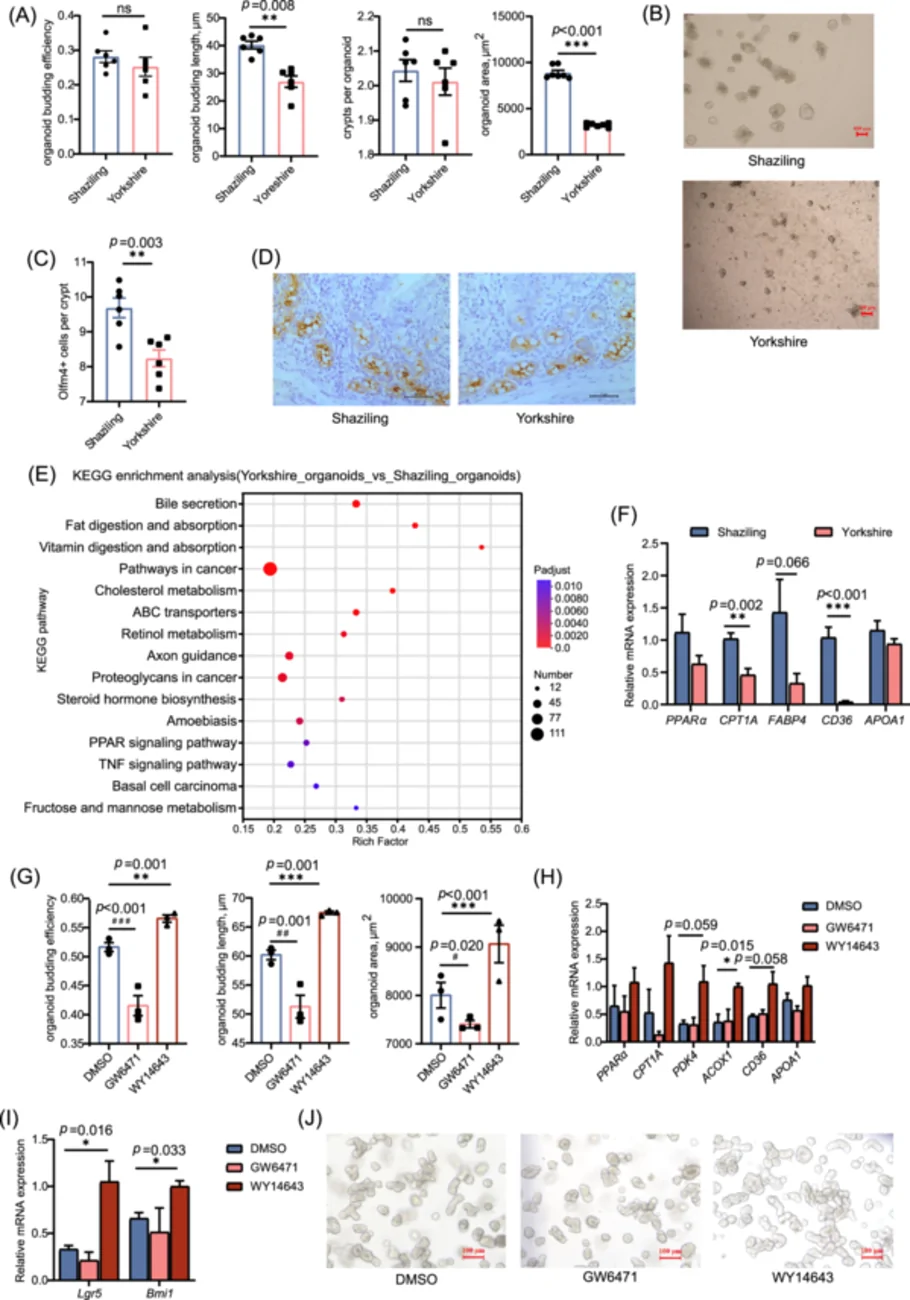
PPARα signaling pathway was more highly expressed in Shaziling pigs. (A) Morphological comparison of organoid formation by intestinal stem cells at the base of crypts in Shaziling and Yorkshire pigs, including budding efficiency, budding length, crypts per organoid, growth area, and (B) the representative images of organoids (*n* = 4 biological replicates/group). (C, D) Immunostaining and quantification of Olfm4+ stem (*n* = 50 crypts from Shaziling and Yorkshire, 6 individuals per group). (E) The KEGG pathway enrichment analysis between the organoids of Shaziling and Yorkshire pigs. (F) The mRNA relative expression of PPARα target gene (*n* = 6 biological replicates/group). (G) The budding efficiency, the budding length, and the growth area of organoids when incubated with DMSO, 5 μM GW6471 and 60 μM WY14643, * used to indicate the difference between the DMSO and WY14643 group, and **^#^** used to indicate the difference between the DMSO and GW6471 group (*n* = 3 biological replicates/group). The mRNA relative expression of PPARα target gene (H), ISC marker, (I) cultured with the indicated treatments for 3 days. (J) The representative images of organoids when incubated with DMSO, 5 μM GW6471 and 60 μM WY14643 (scale bar, 100 μm). KEGG, Kyoto Encyclopedia of Genes and Genomes; PPAR, peroxisome proliferator‐activated receptors; *CD36*, cluster of differentiation 36; *APOA1*, apolipoprotein A1; *CPT1A*, Carnitine Palmitoyltransferase 1A; *PPARα*, Peroxisome proliferator‐activated receptor alpha; *PDK4,* pyruvate dehydrogenase kinase 4; *ACOX1*, acyl‐Coenzyme A oxidase 1; *Lgr5*, leucine‐rich repeat containing G protein coupled receptor 5; *Bmi1*, BMI1 proto‐oncogene, polycomb ring finger. For comparisons between two groups, statistical significance was determined using an unpaired two‐tailed Student's *t*‐test. Data are presented as means ± SEM. Significant differences marked as* for *p* < 0.05, ** for *p* < 0.01, *** for *p* < 0.001.

### Oleoylethanolamine (OEA) produced by lipid metabolism is more enriched in Shaziling pigs

Then, we sought to explore the potential link between altered fatty acid metabolism and PPARα expression. Jejunal lipid metabolomics was investigated using liquid chromatography‐mass spectrometry (LC‐MS), Shaziling and Yprkshire pigs could be separated from each other by PCA analysis and the expression patterns of the samples were similar within each group (Figure 3A). The top 50 differential metabolites in terms of abundance were selected for clustering analysis using variable importance in the projection (VIP) 1, *p* < 0.05 as the standard, of which 25 metabolites were up‐regulated and 25 were down‐regulated in Shaziling pigs (Figure 3B). KEGG enrichment analysis showed that the differential metabolites clustered mainly in glycerophospholipids, linoleic acid, sphingolipids, glycerolipids, and alpha linolenic acid metabolism, as well as in the phospholipase D signaling pathway, which was consistent with the intestinal differential gene KEGG‐enriched pathway, with the most significant effect on glycerophospholipids pathway (Figure 3C). Among the up‐regulated metabolites, we identified NAE (18:1), also known as oleoylethanolamine, or OEA for short (Figure 3D), which was shown to be an endogenous agonist of PPARα. Correlation and chord diagrams showed that OEA and phosphatidylethanolamine (PE) had similar expression patterns and their functions were positively correlated (Figure 3E).

**FIGURE 3:**
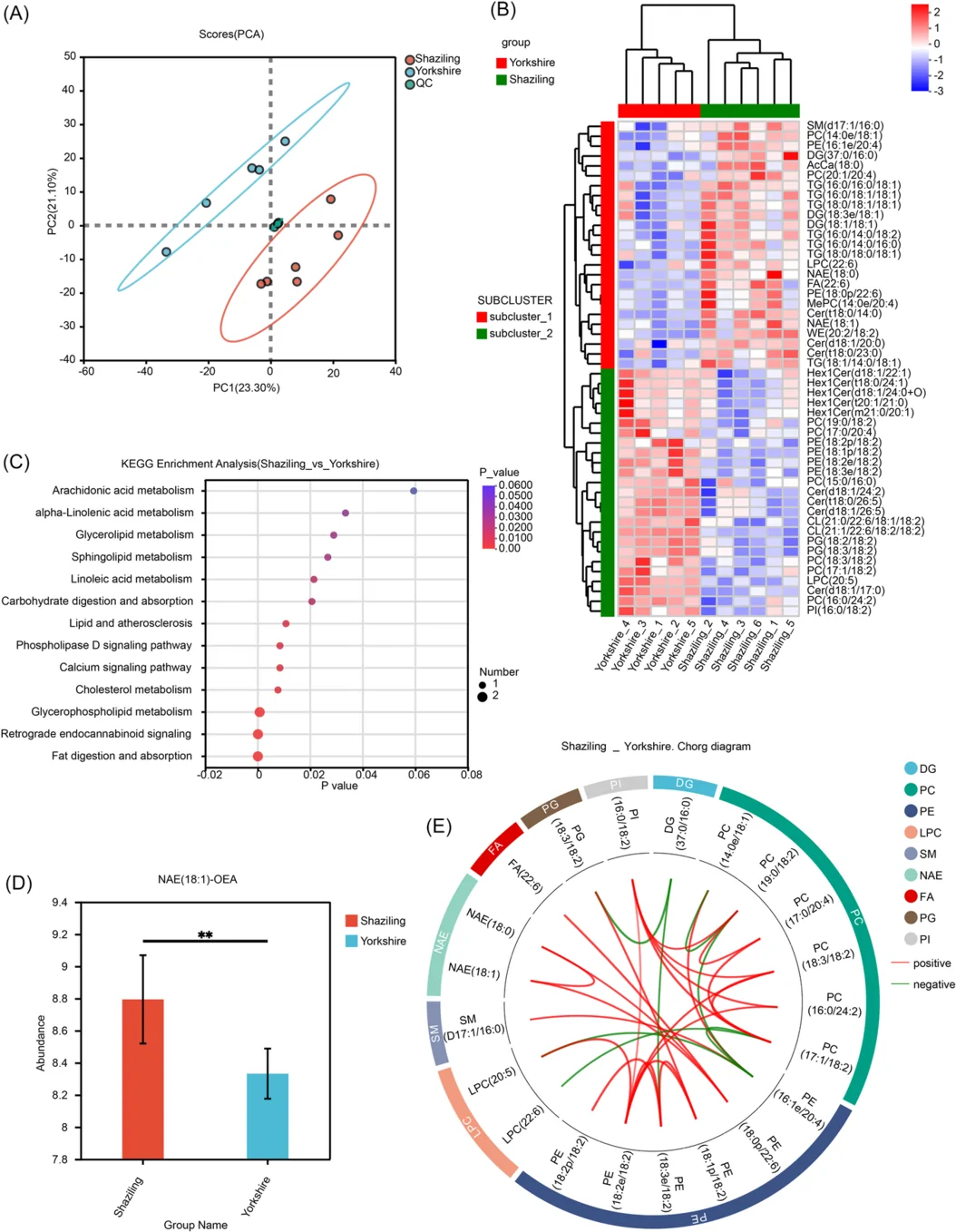
Analysis of lipid metabolism in the jejunum between Shaziling and Yorkshire pigs at 210 day of age. (A) Plot of PCA principal component analysis Yorkshire pigs *n* = 5, Shaziling pigs *n* = 6. (B) Clustering heatmap of differential metabolites. (C) KEGG pathway enrichment map of differential metabolites. (D) Abundance of the metabolite NAE (18:1)‐OEA in the jejunum. (E) Correlation and chord diagrams for different lipid metabolites. PCA: Principal Component Analysis, PC: Principal Component, QC: Quality Control, SM (d17:1/16:0): Sphingomyelin (d17:1/16:0), PC (14:0e/18:1): Phosphatidylcholine (14:0 ether/18:1), PE (16:1e/20:4): Phosphatidylethanolamine (16:1 ether/20:4), DG (37:0/16:0): Diacylglycerol (37:0/16:0), AcCa (18:0): Stearoylacetylcarnitine, PC (20:1/20:4): Phosphatidylcholine (20:1/20:4), TG (16:0/16:0/18:1): Triacylglycerol (16:0/16:0/18:1), TG (16:0/18:1/18:1): Triacylglycerol (16:0/18:1/18:1), TG (18:0/18:1/18:1): Triacylglycerol (18:0/18:1/18:1), DG (18:3e/18:1): Diacylglycerol (18:3 ether/18:1), DG (18:1/18:1): Diacylglycerol (18:1/18:1), TG (16:0/14:0/18:2): Triacylglycerol (16:0/14:0/18:2), TG (16:0/14:0/16:0): Triacylglycerol (16:0/14:0/16:0), TG (18:0/18:0/18:1): Triacylglycerol (18:0/18:0/18:1), LPC (22:6): Lysophosphatidylcholine (22:6), NAE (18:0): N‐Stearoylethanolamine, FA (22:6): Docosahexaenoic Acid, PE (18:0p/22:6): Phosphatidylethanolamine (18:0 plasmalogen/22:6), MePC (14:0e/20:4): Methylphosphatidylcholine (14:0 ether/20:4), Cer (t18:0/14:0): Ceramide (t18:0/14:0), NAE (18:1): N‐Oleoylethanolamine, WE (20:2/18:2): Wax ester (20:2/18:2), Cer (d18:1/20:0): Ceramide (d18:1/20:0), Cer (t18:0/23:0): Ceramide (t18:0/23:0), TG (18:1/14:0/18:1): Triacylglycerol (18:1/14:0/18:1), Hex1Cer (d18:1/22:1): Monohexosylceramide (d18:1/22:1), Hex1Cer (t18:0/24:1): Monohexosylceramide (t18:0/24:1), Hex1Cer (d18:1/24:0 + O): Monohexosylceramide (d18:1/24:0 hydroxylated), Hex1Cer (t20:1/21:0): Monohexosylceramide (t20:1/21:0), Hex1Cer (m21:0/20:1): Monohexosylceramide (m21:0 methylated/20:1), PC (19:0/18:2):Phosphatidylcholine (19:0/18:2), PC (17:0/20:4): Phosphatidylcholine (17:0/20:4), PE (18:2p/18:2): Phosphatidylethanolamine (18:2 plasmalogen/18:2), PE (18:1p/18:2): Phosphatidylethanolamine (18:1 plasmalogen/18:2), PE (18:2e/18:2): Phosphatidylethanolamine (18:2 ether/18:2), PE (18:3e/18:2): Phosphatidylethanolamine (18:3 ether/18:2), PC (15:0/16:0): Phosphatidylcholine (15:0/16:0), Cer (d18:1/24:2): Ceramide (d18:1/24:2), Cer (t18:0/26:5): Ceramide (t18:0/26:5), Cer (d18:1/26:5): Ceramide (d18:1/26:5), CL (21:0/22:6/18:1/18:2): Cardiolipin (21:0/22:6/18:1/18:2), CL (21:1/22:6/18:2/18:2): Cardiolipin (21:1/22:6/18:2/18:2), PG (18:2/18:2): Phosphatidylglycerol (18:2/18:2), PG (18:3/18:2): Phosphatidylglycerol (18:3/18:2), PC (18:3/18:2): Phosphatidylcholine (18:3/18:2), PC (17:1/18:2): Phosphatidylcholine (17:1/18:2), LPC (20:5): Lysophosphatidylcholine (20:5), Cer (d18:1/17:0): Ceramide (d18:1/17:0), PC (16:0/24:2): Phosphatidylcholine (16:0/24:2), PI (16:0/18:2): Phosphatidylinositol (16:0/18:2), KEGG: Kyoto Encyclopedia of Genes and Genomes.

### OEA regulates ISC activity and organoid budding via PPARα signaling pathway

Phosphatidylethanolamine (PE), a subclass of glycerophospholipids, features ethanolamine as its head group [17]. In animal tissues, N‐acyl‐phosphatidylethanolamine (NAPE) is biosynthesized through the transfer of an acyl group to the ethanolamine head group of PE. This reaction is catalyzed by either Ca²⁺‐dependent N‐acyltransferases (cPLA2β) [18] or Ca²⁺‐independent N‐acyltransferases (designated as PLATT1‐5) [19]. Subsequently, NAPE undergoes hydrolysis catalyzed by NAPE‐specific phospholipase D (NAPE‐PLD), yielding N‐acylethanolamines (NAEs) and phosphatidic acid as the reaction products(Figure 4A) [20].

**FIGURE 4:**
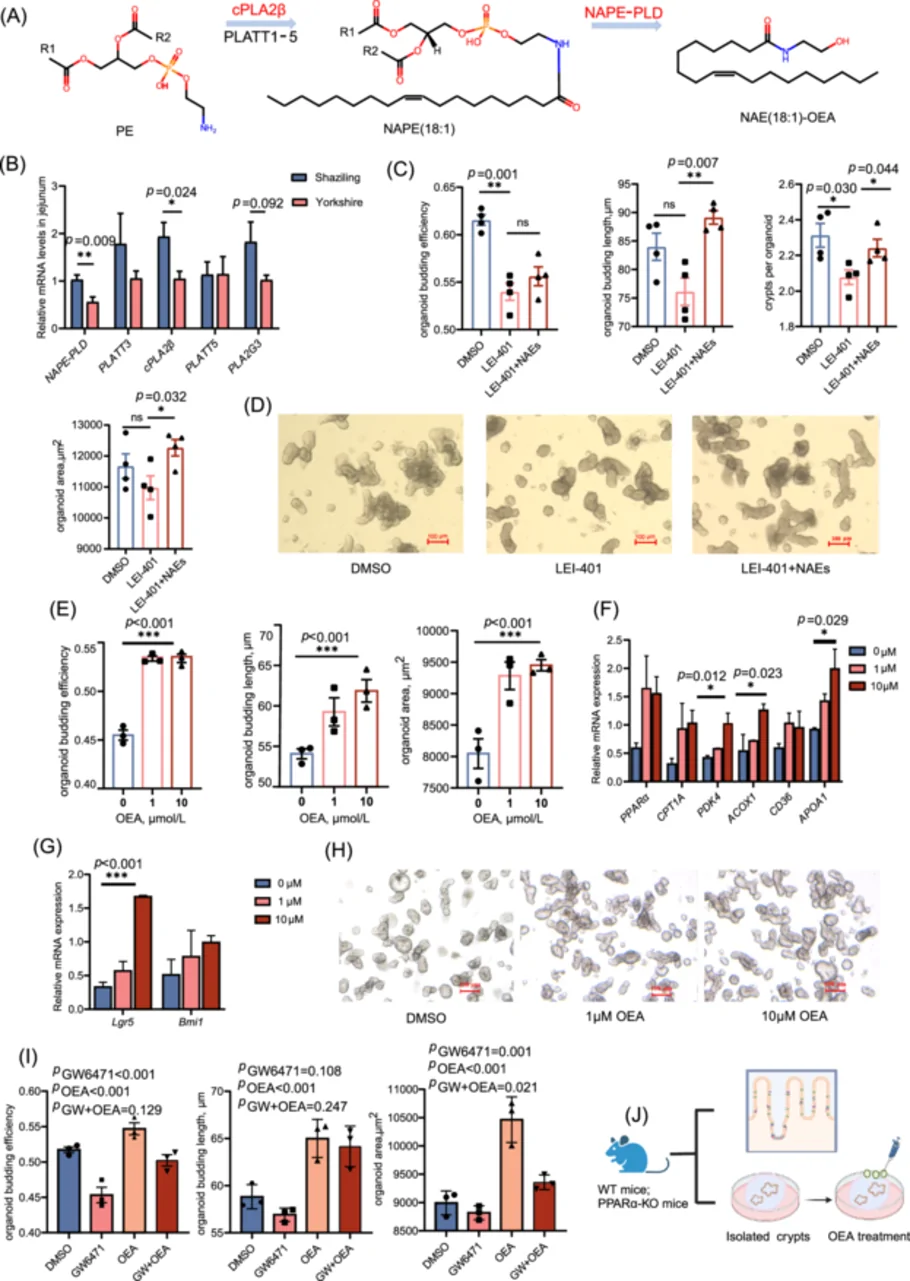
OEA regulates intestinal stem cell activity and organoid budding via PPARα signaling pathway. (A) Schematic representation of the synthesis from phosphatidylethanolamine (PE) to NAE (18:1)‐OEA in animal cells. (B) The mRNA relative expression of NAPEs and NAEs‐related synthetic and degradation genes in 210‐day‐old Shaziling and Yorkshire pigs (*n* = 6 biological replicates/group). (C) Morphological analysis of organoids when incubated with DMSO, 5 μM LEI‐401 and 5 μM LEI‐401 + 10 μM NEAs, including budding efficiency, budding length, crypts per organoid, growth area of organoids and (D) the representative images of organoids (the NAEs contained 10 µM OEA and 10 µM PEA, scale bar, 100 μm, *n* = 4 biological replicates/group). (E) The budding efficiency, the budding length and the growth area of organoids when incubated with DMSO, 1 μM and 10 μM OEA (*n* = 3 biological replicates/group). The mRNA relative expression of PPARα target gene (F), ISC marker (G), cultured with the indicated treatments for 3 days. (H) The representative images of organoids when incubated with DMSO, 1 μM and 10 μM OEA (scale bar, 100 μm). (I) The budding efficiency, the budding length and the growth area of organoids when incubated with DMSO, 5 μM GW6471, 10 μM OEA and 5 μM GW6471 + 10 μM OEA (*n* = 3 biological replicates/group). (J) WT and PPARα‐KO mice were selected to analyze their intestinal morphology and isolate intestinal organoids for OEA treatment. WT, wild‐type; PE, Phosphatidylethanolamine; NAPE, N‐acylphosphatidylethanolamine; NAEs,: N‐acylethanolamines; *NAPE‐PLD*, N‐acyl phosphatidylethanolamine phospholipase D; *FAAH*, fatty acid amide hydrolase; *NAAA*, N‐acylethanolamine acid amidase; *PLATT3*, phospholipase A and acyltransferase 3; *PLATT5*, phospholipase A and acyltransferase 5; *cPLA2β*, phospholipase A2 group IVB; *PLA2G3*, phospholipase A2 group III; *CD36*, cluster of differentination 36; *APOA1*, apolipoprotein A1; *CPT1A*, Carnitine Palmitoyltransferase 1A; *PPARα*, Peroxisome proliferator‐activated receptor alpha; *PDK4,* pyruvate dehydrogenase kinase 4; *ACOX1*, acyl‐Coenzyme A oxidase 1; *Lgr5*, leucine‐rich repeat containing G protein‐coupled receptor 5; *Bmi1*, BMI1 proto‐oncogene, polycomb ring finger. For comparisons between two groups, statistical significance was determined using an unpaired two‐tailed Student's *t*‐test. For comparisons among three or more independent groups under a single‐factor design, a one‐way ANOVA was performed, followed by Duncan's multiple range test for post hoc analysis if the overall *F*‐test was significant. For two‐way ANOVA, when a significant interaction or main effect was detected, post hoc multiple comparisons were conducted. Specifically, for the detailed exploration of all pairwise differences following a significant interaction, Fisher's Least Significant Difference (LSD) test was applied. If a significant main effect was found, Duncan's new multiple range test was employed for multiple comparisons. Data are presented as means ± SEM. Significant differences marked as* for *p* < 0.05, ** for *p* < 0.01, *** for *p* < 0.001.

OEA, a naturally occurring lipid mediator synthesized in the small intestine, belongs to the NAEs family [21]. Elevated *cPLA2β* and *NAPE‐PLD* expression in 210‐day‐old Shaziling pigs partially corroborates OEA's role in intestinal development (Figure 4B). Pharmacological inhibition of *NAPE‐PLD* with LEI‐401 in organoids suppressed NAE synthesis and significantly impaired growth (Figure 4B), supporting our hypothesis. Subsequent NAE supplementation restored organoid budding length (Figure 4C,D), crypts per organoid (Figure 4C,D), and growth area (Figure 4C,D), further confirming the important role of NAEs for intestinal development.

Then, organoids were incubated with the OEA and compared to controls. As expected, OEA treatment increased organoid budding rate, budding length, and growth area in a dose‐dependent manner (Figure 4E). Quantitative RT‐PCR confirmed elevated expression of PPARα target genes (Figure 4F) and ISC markers *Lgr5* and *Bmi1* (Figure 4G). Co‐treatment with GW6471 and OEA revealed a certain interaction, further supporting that OEA affects the growth of intestinal organoids via PPARα (Figure 4H,I). These findings suggest that OEA activation of PPARα enhances ISC renewal, as evidenced by increased budding length and ISC marker expression.

### Regulation of ISC activity by OEA may be mediated through oxidative phosphorylation

The role of OEA was further explored using genetic mouse models (Figure 4J). Comparative analysis of intestinal morphology in wild‐type (WT) and Peroxisome proliferator‐activated receptor alpha‐knockout (PPARα‐KO) mice revealed reduced VH in PPARα‐KO mice (Figure 5A,B), indicating the role of PPARα in villus development. Jejunal crypts isolated from both genotypes were cultured. OEA supplementation enhanced budding efficiency, budding length, and growth area in WT‐derived organoids (Figure 5C). However, these effects were abolished in PPARα‐KO organoids (Figure 5D), which further proved the effect of OEA on the PPARα target activation.

**FIGURE 5:**
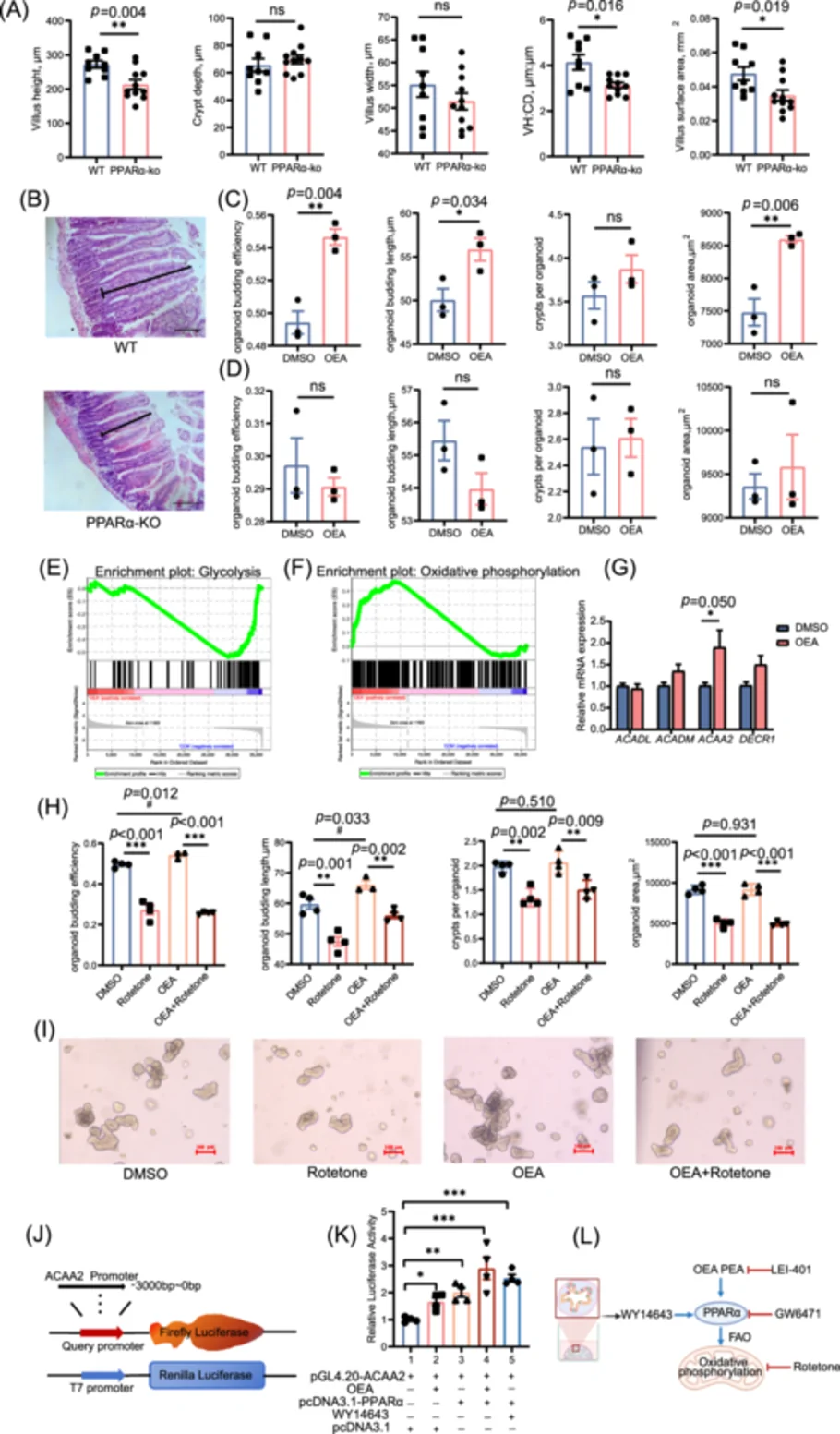
OEA enhances oxidative phosphorylation via PPARα, thereby promoting organoid budding and intestinal stem cell differentiation. (A) Average jejunum villus height, crypt depth, villus width, VH:CD and villus surface area, from *n* = 9 mice in group WT and *n* = 11 mice in group PPARα‐KO. (B) Representative H&E staining in mice jejunum, scale bar = 100 μm. (C) The budding efficiency, the budding length, crypts per organoid, and growth area of intestinal organoids in wild‐type mice incubated with DMSO and 10 μM OEA (*n* = 3 biological replicates/group). (D) The budding efficiency, the budding length, crypts per organoid, and growth area of intestinal organoids in PPARα‐KO mice incubated with DMSO and 10 μM OEA (*n* = 3 biological replicates/group). (E) The gene set enrichment analysis of Glycolysis in organoids cultured with OEA treatment. (F) The gene set enrichment analysis of Oxidative phosphorylation in organoids cultured with OEA treatment. (G) The mRNA relative expression of PPARα and oxidative phosphorylation target gene in organoids cultured with OEA treatment (*n* = 4 biological replicates/group). (H,I) Morphological analysis of organoids when incubated with DMSO, 0.05 μM Rotetone, 10 μM OEA and 0.05 μM Rotetone + 10 μM OEA, including budding efficiency, budding length, crypts per organoid, growth area of organoids, and (I) The representative images of organoids (*n* = 4 biological replicates/group. (scale bar, 100 μm). The asterisk * indicates a comparison between the DMSO and the Rotetone as well as the OEA and the OEA + Rotetone, while ^#^ indicates a comparison between the DMSO and the OEA. (J) Schematic diagram of dual‐luciferase reporter vector construction for the ACAA2 promoter. (K) PPARα overexpression plasmid pcDNA3.1‐PPARα (800 ng) or vector plasmid as a control pcDNA 3.1 (800 ng) was co‐transfected into 293T cells along with pGL4.20‐ACAA2 (400 ng) and pRL‐TK (40 ng) internal reference plasmids. The transfected cells were treated with ligands (5 μM OEA or 60 μM WY14643) after 24 h and the cells were used for reporter gene assay after 24 h. Values are expressed as fold induction of the control (values when only transfected with the reporter plasmid pGL4.20‐ACAA2 and vector plasmid pcDNA 3.1) and are set to 1 (*n* = 4 biological replicates/group). (L) Graphical representation of the mechanism of PPARα: Agonism (WY14643, OEA, PEA) and antagonism (GW6471) of PPARα and the pharmacological effects of LEI‐401, an inhibitor of OEA and PEA synthesis, and rotenone, an inhibitor of oxidative phosphorylation. VH:CD, villus height to crypt depth. *ACADL*, acyl‐Coenzyme A dehydrogenase, long‐chain; *ACADM*, acyl‐CoA dehydrogenase medium chain; *ACAA2*, acetyl‐CoA acyltransferase; *DECR1*, 2,4‐dienoyl‐CoA reductase 1. For comparisons between two groups, statistical significance was determined using an unpaired two‐tailed Student's *t*‐test. Data are presented as means ± SEM. Significant differences marked as* for *p* < 0.05, ** for *p* < 0.01, *** for *p* < 0.001.

Transcriptomic profiling of OEA‐treated organoids revealed downregulated glycolysis pathways (Figure 5E) and upregulated Oxidative phosphorylation (OXPHOS) activity (Figure 5F). OEA treatment enhanced the expression of PPARα and OXPHOS target genes (Figure 5G). Organoid modeling demonstrated that mitochondrial OXPHOS inhibition via respiratory chain blockade reduced crypt formation and partially attenuated OEA effects to a certain extent (Figure 5H,I), suggesting OEA promotes ISC proliferation and differentiation through increasing OXPHOS. To determine that one of OXPHOS target genes, *ACAA2*, is directly regulated by PPARα [22], we performed dual‐luciferase reporter assays. The peroxisome proliferator‐responsive elements (PPREs) of PPARα have been reported to be conserved among species [23]. We examined the 3 kb upstream of the *ACAA2* transcription start site in human, mouse, and pig. For each putative PPRE identified, the predicted PPARα‐specific binding strength was determined, and at least one PPRE was conserved among pig, mouse, and human (Figure S4). The −3000–0 bp fragment of the human *ACAA2* promoter was cloned upstream of the pGL4.20 luciferase reporter gene to construct a reporter plasmid (Figure 5J). We co‐transfected PPARα overexpression plasmid (pcDNA3.1‐PPARα) or vector plasmid (pcDNA 3.1) as a control with pGL4.20‐ACAA2 and pRL‐TK internal reference plasmids into 293 T cells, which were subsequently incubated with ligands (5 µM OEA, 60 µM WY14643) or dimethyl sulfoxide (DMSO) for 24 h. The results showed that PPARα overexpression induced the promoter activity of ACAA2 onefold higher than the control (Figure 5K). In contrast, OEA‐activated PPARα induced the promoter activity of *ACAA2* twofold higher than the control, indicating that PPARα can directly regulate *ACAA2* promoter activity (Figure 5K). Collectively, these findings demonstrate that OEA enhances OXPHOS via PPARα, thereby promoting ISC proliferation and differentiation and organoid budding.

### Dietary OEA and PEA improved intestinal villus size and nutrient digestibility

Comparative analysis of intestinal morphology between Shaziling and Yorkshire pigs revealed that elevated levels of OEA in the intestines of Shaziling pigs correlated with increased VH. To further test this idea, we fed OEA or Palmitoylethanolamine (PEA) − another endogenous PPARα ligand to ([Yorkshire×Landrace] × Duroc) piglets. To investigate whether the number of budding new crypts from the developed organoid is proportional to the intestine surface size in vivo, crypts were isolated from the mid‐small intestine of control and treated piglets. ISC from OEA‐ and PEA‐treated groups exhibited superior organoid formation, budding capacity (Figure 6A,B, Figure S5A,B), and larger organoid area (Figure 6A,B, Figure S5A,B), and the second‐generation organoids in the PEA group also had stronger budding ability (Figure S5C,D). OEA or PEA treatment significantly increased VH, CD, VW, villus surface area (VSA), and VH:CD ratio in the mid‐small intestine compared to controls (Figure 6C,D, Figure S5E). Proliferation and differentiation assays demonstrated that OEA significantly increased Ki67‐positive cells (Figure 6E), endocrine cells (Figure 6F,G), and goblet cells (Figure 6H,I) compared to controls, with representative images shown in Figure 6J.

**FIGURE 6:**
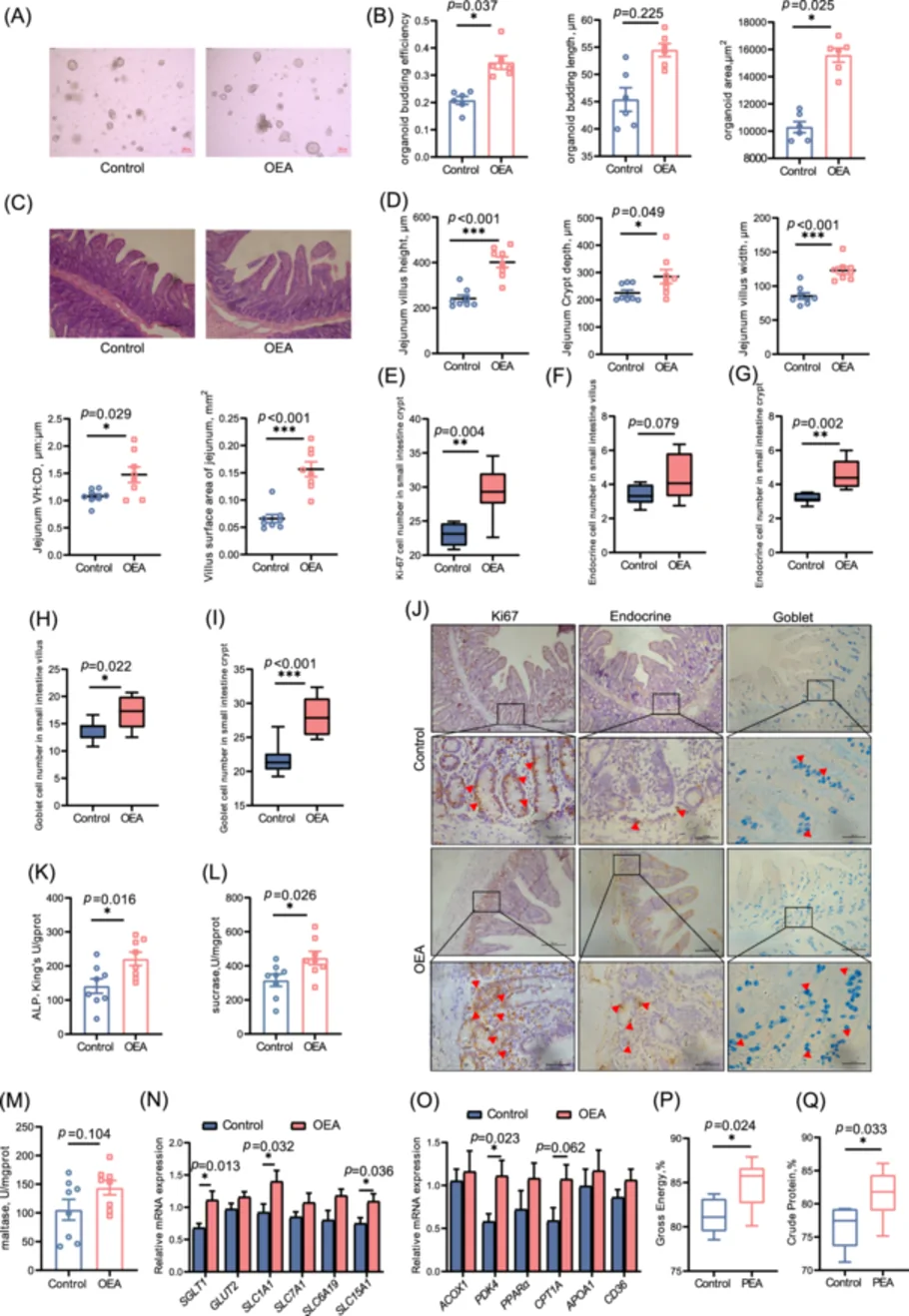
Dietary OEA and PEA improved intestinal morphology and nutrient digestibility. (A) The representative images of primary organoids in control and OEA group (scale bar, 100 μm). (B) Morphological comparison of organoid formation by intestinal stem cells at the base of crypts in control and OEA piglets, including budding efficiency, budding length, and growth area of organoids (*n* = 6 biological replicates/group). (C) The representative images of the middle small intestine morphology in the control and OEA group (magnification 100×; scale bar, 200 μm). (D) The villus height, crypt depth, villus width, VH:CD and villus surface area of the middle small intestine. (E) The number of Ki67‐positive cells in the middle small intestine crypt. (F,G) The number of endocrine cells in the middle small intestine villus and crypt. (H,I) The number of goblet cells in the middle small intestine villus and crypt. (J) The representative images of Ki67‐positive cells, endocrine cells, and goblet cells in the control and OEA group, Ki67 and endocrine cells are dark brown, and goblet cells are blue, they are marked by red triangles (magnification 100×; scale bar, 200 μm and magnification 400×; scale bar, 50 μm). (K) The ALP activity. (L) The sucrase activity. (M) The maltase activity. (N) The mRNA relative expression of nutrient transporter in control and OEA group. (O) The mRNA relative expression of PPARα target gene in control and OEA group. (P) The apparent total tract digestibility of whole‐gut gross energy between control and PEA group. (Q) The whole‐gut apparent total tract digestibility of crude protein between control and PEA group. ALP, alkaline phosphatase; VH:CD, villus height to crypt depth; *SGLT1*, sodium glucose cotransporter 1; *GLUT2*, recombinant glucose transporter 2; *SLC6A19*, solute carrier family 6 member 19; *SLC7A1*, solute carrier family 7 member 1; *SLC1A1*, solute carrier family 1 member 1; *SLC15A1*, solute carrier family 15 member 1; *CD36*, cluster of differentination 36; *APOA1*, apolipoprotein A1; *CPT1A*, Carnitine Palmitoyltransferase 1A; *PPARα*, Peroxisome proliferator‐activated receptor alpha; *PDK4*, pyruvate dehydrogenase kinase 4; *ACOX1*, acyl‐Coenzyme A oxidase 1. For comparisons between two groups, statistical significance was determined using an unpaired two‐tailed Student's *t*‐test. Data are presented as means ± SEM (*n* = 8 biological replicates/group). Significant differences marked as* for *p* < 0.05, ** for *p* < 0.01, *** for *p* < 0.001.

Brush border enzyme activity analysis revealed higher alkaline phosphatase and sucrase activities in OEA‐treated piglets (Figure 6K,L), while there was no difference in maltase (Figure 6M). Additionally, OEA upregulated the expression of nutrient transporters (*SGLT1*, *SLC1A1*, *SLC15A1*; Figure 6N), which also indicated that stem cell proliferation and differentiation are closely related to intestinal absorption. Our results also showed that OEA and PEA enhanced the expression of *PPARα* target genes at the transcriptional level (Figure 6O, Figure S5F), without affecting the expression of *PPARγ* or *PPARδ* (Figure S5G,H). It is worth noting that the addition of PEA improved energy and crude protein digestibility (Figure 6P,Q). Collectively, dietary supplementation with endogenous lipid signaling molecules OEA or PEA can improve intestinal morphology and nutrient digestibility via PPARα.

## 3 DISCUSSION

Pigs serve as an important source of animal protein for humans and hold substantial value in agricultural production [24]. Against the backdrop of global reduction in arable land and the need for efficient utilization of feed resources, feed efficiency has become a critical trait in animal breeding due to its significant economic and ecological impacts [25]. Here, by investigating the changes in lipid metabolism in ISCs and their impact on villus development, we identified an important endogenous lipid signaling molecule— OEA and elucidated its mechanistic role in intestinal morphological development.

Our results demonstrate that obese‐type Shaziling pigs and lean‐type Yorkshire pigs employ distinct adaptive mechanisms for intestinal nutrient digestion and absorption. Intestinal function is closely linked to its histomorphology. Increased VH expands the absorptive surface area, thereby enhancing nutrient absorption capacity [26]. Both Shaziling and Yorkshire pigs exhibit a significant increase in VH with age, which expands the absorptive surface area, leading to improved digestion and absorptive capacity. The VH:CD ratio serves as a criterion for assessing the digestive and absorptive capacity of the small intestine. Shaziling pigs across various ages displayed a higher VH:CD ratio compared to Yorkshire pigs. Research by Pluske et al. suggests that pigs with longer villi possess a greater number of mature enterocytes expressing maximal digestibility, resulting in heightened sucrase activity on the villi [27]. Our findings are consistent with existing research, demonstrating that Shaziling pigs exhibit longer villi and higher sucrase activity. Intestinal alkaline phosphatase is a pivotal marker enzyme for changes in the digestive and absorptive functions of the small intestine. It is expressed and secreted by intestinal epithelial cells and is highly expressed on the small intestinal mucosa [28], closely associated with protein and lipid metabolism [29]. Upon entering the small intestine, proteins, sugars, and fats are degraded into amino acids, monosaccharides, and fatty acids, respectively, and absorbed by enterocytes through corresponding transporters. The alkaline phosphatase activity in the jejunal mucosa of Shaziling pigs peaked at 90 days of age, and the expression of nutrient transporters such as *SLC6A19*, *SLC7A1*, and *FATP4* was also elevated, indicating superior absorption of proteins and lipids. Furthermore, our study found that the gene expression levels of *SGLT1* and *GLUT2* in Shaziling pigs of different ages were higher than those in Yorkshire pigs. RNA sequencing analysis further revealed that Shaziling pigs outperformed Yorkshire pigs in fat, vitamin, and mineral digestion and absorption. However, the length and weight of the small intestine in Yorkshire pigs were significantly greater than those in Shaziling pigs, which also increased the epithelial area to some extent, potentially offsetting differences in villus morphology, digestive enzyme activity, and transporter carrier expression. At the macroscopic level, the digestive and absorptive capacities of the two breeds were comparable, as confirmed by our digestibility measurements. As anticipated, no significant difference in digestibility was observed between the two breeds. In summary, Shaziling and Yorkshire pigs employ distinct adaptive mechanisms for the digestion and absorption of intestinal nutrients.

Enhanced digestive enzyme activity may supply additional energy and substrates for ISCs, thereby promoting their proliferation and differentiation, which are crucial for the formation and maintenance of intestinal villus [30]. The associated proliferative activity can be evaluated by quantifying Ki67‐positive cells residing in the proliferative zone of the crypts and the base of the villi [31]. The quantity of Ki67‐positive cells in pig crypts significantly decreases with increasing age, potentially due to reduced epithelial cell proliferation rates, slower migration of epithelial cells from crypts to villus tips, or epithelial cell apoptosis and increased shedding rates [32]. Studies have shown that the overall proliferation rate of epithelial cells during crypt morphogenesis in mice is significantly higher than that in adult mice, which indicates that the cell proliferation rate will gradually decrease with the maturation of intestinal epithelium [33]. Concurrently, RNA sequencing revealed significant differences in the enrichment of lipid metabolic pathways between the two pig breeds. Notably, lipid metabolism pathways are associated with ISC. Lipid metabolism not only provides energy to intestinal cells but also influences the proliferation and differentiation of ISCs by activating specific signaling pathways, such as the PPARα pathway [34]. Comparative analyses of intestinal development, organoid development, and transcriptional differences revealed breed‐specific divergence in lipid metabolism, particularly enrichment in distinct fatty acid metabolic pathways and the PPAR signaling pathway. Among the three PPAR subtypes (α, γ, δ), PPARα and Peroxisome proliferator‐activated receptor delta (PPARδ) are predominantly expressed in the intestine, with PPARα showing peak abundance in the mid‐small intestine [35]. GSEA further identified that the target was enriched and that these genes were relatively highly expressed in Shaziling pigs. Upon exploring the role of PPARα in regulating the small intestinal epithelial development, we discovered that PPARα activation significantly promoted the differentiation of crypt stem cells. Specifically, PPARα activation stimulated stem cells differentiation in the small intestinal crypts, a critical step in the process of intestinal epithelial renewal and repair.

Nuclear receptors are ligand‐activated transcription factors that bind to regulatory regions of target genes to regulate their expression [36]. Consequently, these receptors enable organisms to integrate environmental signals and adapt by modulating the expression levels of relevant genes. As a key member of the nuclear receptor family, PPARα can be activated by fatty acids, diverse lipid species, and a class of chemicals termed peroxisome proliferators [37]. Using LC‐MS, we systematically profiled lipid metabolites in the intestines of Shaziling and Yorkshire pigs, identifying major classes including glycerophospholipids, sphingolipids, glycerolipids, sterol lipids, and fatty acyls. Among fatty acyl lipids, NAEs constitute a family of bioactive fatty acid amides with diverse roles in inflammation, neurotransmission, appetite regulation, fertility, stress, and anxiety. The signaling functions of NAEs are determined by their structural incorporation of saturated, mono‐ or polyunsaturated fatty acyl chains. Among upregulated metabolites, we identified NAE (18:0) (stearoylethanolamide, SEA) and NAE (18:1) (oleoylethanolamide, OEA), both demonstrating anti‐inflammatory properties. However, unlike OEA, SEA exhibits no affinity for PPARα [21]. Further experiments revealed that OEA‐mediated promotion of intestinal organoid growth and stem cell viability is critically dependent on PPARα. The OEA‐induced organoid growth effect was attenuated by treatment with the synthetic PPARα inhibitor GW6471; whereas in the PPARα‐KO mice model, the regulatory effect of OEA on crypt stem cell growth was completely counteracted. Additionally, PPARα deficiency resulted in a significant reduction of intestinal VH in mice, potentially linked to its role in lipid metabolism regulation.

During lipid metabolism, Acylcarnitines transport fatty acids into the mitochondria, where they undergo oxidation to generate energy [34]. The energy released during this process is converted to ATP for cellular use through the process of OXPHOS. Mitochondrial OXPHOS drives cell differentiation and crypt formation. Rodríguez et al. [38] observed that spherical WENR organoids exhibited elevated glycolytic activity, whereas budding organoids drove crypt morphogenesis through enhanced mitochondrial OXPHOS efficiency, aligning with our findings. In our organoid culture model, OEA supplementation significantly increased budding efficiency and budding length, concurrent with elevated OXPHOS levels. Whereas budding was severely inhibited by blocking the electron transport in the respiratory chain, suggesting that the energy metabolism of the cells was affected, and that the increase in the efficiency of OXPHOS directly increased the energy supply of the cells. Furthermore, the bioenergetic surplus generated by OXPHOS may underpin the coordinated expansion of the intestinal epithelium. The observed increase in villus size, both in our models and in Shaziling pigs, necessitates not only enhanced differentiation but also a robust supply of progenitor cells. Our data, revealing a higher abundance of Ki67‐positive cells, points to such heightened proliferative activity. We propose that the OEA‐PPARα‐OXPHOS axis, while a direct driver of differentiation, also creates a metabolic microenvironment conducive to proliferation. The abundant ATP production likely lowers the metabolic barriers to cell cycle progression, thereby facilitating the proliferation of intestinal stem and progenitor cells. As a high‐affinity PPARα agonist, OEA regulates lipid metabolism. *CD36*, *ACOX1*, and *CPT1A* are involved in this process and are directly regulated by PPARα [39]. We observed higher expression levels of these genes in piglets fed OEA, which also demonstrated the activation of PPARα in piglets. While prior studies emphasize that OEA exerts its anti‐inflammatory effects through PPARα [40], our work reveals that PPARα activation by OEA promotes intestinal nutrient absorption and epithelial development, possibly because energy generated from lipid metabolism promotes ISC proliferation and differentiation to increase the number of mature intestinal epithelial absorptive cells, which needs to be further investigated. Notably, PEA—another member of the NAE family has been established as an endogenous ligand of PPARα [41]. Our findings demonstrate that PEA exhibits comparable bioactivity to OEA, with dietary PEA supplementation significantly enhancing crude protein and energy digestibility in piglets. Furthermore, the inherent safety profile of NAEs is ensured through their rapid hydrolysis by fatty acid amide hydrolase [42] or N‐acylethanolamine acid amidase [43] into ethanolamine and corresponding fatty acids, effectively preventing toxic metabolite accumulation and thus ensuring their safe oral bioavailability. However, this study has several limitations. First, the regulatory role of OEA was investigated under standard physiological conditions. Future studies are necessary to explore its function and efficacy in challenged states, such as during metabolic stress or enteric disease. Second, the dietary supplementation experiment was conducted using weaned piglets as the model. Whether OEA exerts similar beneficial effects on ISC activity in growing—finishing pigs remain to be determined and represents an important direction for subsequent research.

## 4 CONCLUSION

In summary, lipid metabolism is involved in the regulation of intestinal villus size. Specifically, in Shaziling pigs, OEA derived from lipid metabolism targets and activates the PPARα signaling pathway, thereby enhancing the proliferation and differentiation capacity of ISCs (Figure S6). This leads to an increase in the size of intestinal villi, which further expands the digestive and absorptive surface area and improves functional efficiency. Consequently, this adaptation compensates for the relatively shorter small intestine length in Shaziling pigs, enabling their digestive efficiency to remain comparable to that of the lean‐type commercial breed, Yorkshire pigs. Furthermore, Exogenous supplementation of PEA improves the nutrient digestibility of weaned piglets in commercial breeds. Future investigations should focus on elucidating the structure‐activity relationship between NAE structural features (e.g., acyl chain saturation) and receptor selectivity to enhance their applicability in nutritional interventions.

## 5 METHODS

### Animals and experimental treatments

Experiment 1: Shaziling pigs were procured from Xiangtan Xinxiangnong Ecological Science and Technology Co., Ltd., and Yorkshire pigs were obtained from Taihe County Aomu Breeding Co., Ltd. of Aonong Group. Six Shaziling pigs and six Yorkshire pigs were purchased at each of 30, 60, 90, 150, 210, and 300 days of age for slaughter, with six replicates per age group.

Experiment 2: Sixteen weaned piglets ([Yorkshire×Landrace] × Duroc) with similar body weight (7.45 ± 0.19 kg) were selected to investigate the role of PPARα in the intestine. The piglets were randomly assigned to two groups. The control group was fed a standard diet, while the experimental group received a diet supplemented with 5 mg/kg OEA for four consecutive weeks [44]. The ingredients and nutrient composition of the experimental diet are detailed in Table S1.

Experiment 3: Sixteen weaned piglets ([Yorkshire×Landrace] × Duroc) with similar body weight (7.46 ± 0.26 kg) were selected to examine the role of PPARα in the intestine. The piglets were randomly divided into two groups. The control group was fed a standard diet, while the experimental group received a diet supplemented with 10 mg/kg PEA for four consecutive weeks [45]. The ingredients and nutrient composition of the experimental diet are provided in Table S2.

Experiment 4: The 3‐week‐old PPARα‐KO and WT mice were used in this study. The WT mice (C57BL/6 background) were purchased from Shanghai Model Organisms Company (China). The PPARα‐KO mice were kindly provided by the research group of Prof. Guolin Li from Hunan Normal University. All mice were reared in a specific pathogen‐free barrier facility at the Medical School of Hunan Normal University; mice were fed standard mice chow pellets, had access to tap water supplied in bottles under controlled temperature (23°C), humidity (55% ± 10%), and light (12:12‐h light‐dark cycle) conditions and used in experiments at 6 weeks of age.

### Sample collection and measurements

Shaziling and Yorkshire pigs of the same age were euthanized at 30, 60, 90, 150, 210, and 300 days of age, respectively, according to the method described in the literature [46]. The gastrointestinal tract was removed, and the weight of the stomach, small intestine, and total small intestine length were recorded. The organ index was calculated based on the slaughter weight. Samples of the duodenum, jejunum, and ileum were collected immediately, depleted of intestinal contents, and stored in a 10% formalin solution for morphological analysis. For the jejunum, intestinal segments from both Shaziling and Yorkshire pigs were cut longitudinally along the intestinal tract. After rinsing the intestinal contents, the jejunal mucosa was carefully collected using a glass slide, wrapped in aluminum foil, frozen in liquid nitrogen, and stored at −80°C until analysis. The sample collection method for piglets was identical to that described above.

### Intestinal histomorphology

The duodenum, jejunum, and ileum immersed in a 10% formalin solution were dehydrated using gradient alcohols, cleared with xylene, and then embedded in paraffin to create paraffin blocks [47]. Sections with a thickness of 3–5 μm were cut from the paraffin blocks and stained with hematoxylin and eosin to evaluate the morphology of the small intestine and to prepare for histometric analysis. The VH, CD, and VW of the duodenum, jejunum, and ileum were measured using a microscope with a 100× magnification, combined with an image processing and analysis system (Version 4.12, Leica Imaging Systems Ltd.), and analyzed using Image‐Pro Plus 6.0 software. At least 30 well‐oriented and intact villi and their associated crypts were examined in each intestinal section from the pig. The VH/CD and VSA were calculated. The VSA was determined using the formula VSA = π × VH × VW, as described by Kisielinski's method [48]. The average measurement value of 30 intact and well‐formed villi and crypt structures was used for analysis.

### Immunohistochemistry and goblet cell statistics

A total of 4 μm‐thick tissue sections were cut from paraffin blocks and placed on glass slides for immunohistochemical studies. The sections were dewaxed twice in xylene for 10 min each and then dehydrated in ethanol at decreasing concentrations (100%, 95%, 85%, and 75%) for 5 min each. Following this, the sections were washed in phosphate‐buffered saline (PBS) four times for 3 min each. Endogenous peroxidase activity was blocked using 3% hydrogen peroxide for 10 min in the dark, followed by four PBS washes. The sample portion was then microwaved twice in sodium citrate buffer for antigen retrieval. Next, the sections were incubated with diluted bovine serum albumin in a humidified chamber for 30 min at 37°C. The sections were subsequently incubated with the primary antibody anti‐Ki67 (Abcam, ab15580; dilution 1:800) for 90 min at 37°C. After washing four times with PBS, the sections were incubated with the secondary antibody (ZSGB‐BIO) for 50 min at 37°C. Positive cells were visualized using diaminobenzidine (DAB) (ZSGB‐BIO). The sections were then counterstained with hematoxylin, dehydrated, and mounted. The number of brown‐stained cells was counted in at least 30 crypts per section. For endocrine cell analysis, the primary antibody was replaced with chromogranin A antibody (Abcam, ab45179; dilution 1:700; incubation 90 min at 37°C), while the remaining steps remained unchanged. For ISCs, the primary antibody was replaced with olfm4 antibody (CST, 14369, diluted 1:200, incubation 90 min at 37°C), while the remaining steps remained unchanged. The number of goblet cells was determined using the AB‐PAS Stain Kit (Nanjing Jiancheng Bioengineering Institute).

### Brush border enzyme activity assay

Jejunal mucosal tissue samples from Shaziling and Yorkshire pigs were ground in liquid nitrogen and then mixed with 0.9% normal saline at a volume nine times that of the tissue sample. The mixture was centrifuged (3000 × *g*, 4°C, 10 min), and the supernatant was collected and stored at −80°C until further analysis. The total protein concentration of the supernatant was determined using the Bicinchoninic Acid Protein Assay Kit (Beyotime Biotechnology). The activities of maltase, sucrase, and alkaline phosphatase in the jejunal mucosal supernatants were measured using the Maltase Assay Kit, Sucrase Assay Kit, and Alkaline Phosphatase Assay Kit (Nanjing Jiancheng Bioengineering Institute), respectively, following the manufacturer's instructions.

### Quantitative RT‐PCR

Total RNA was extracted from the jejunal mucosa and organoids using Trizol reagent (TaKaRa). RNA concentration and purity were assessed, and RNA integrity was confirmed by 1% agarose gel electrophoresis, visualizing distinct 28S and 18S rRNA bands. Reverse transcription was performed using the PrimeScript® RT reagent kit with gDNA eraser to remove genomic DNA (42°C for 2 min), followed by the reverse transcription reaction as per the kit instructions (37°C for 15 min and 85°C for 5 s). Primers for this experiment were designed using Primer‐Blast. Primer sequences and lengths for the target genes in jejunal mucosa and organoids are listed in Table S3. For RT‐PCR, cDNA was diluted fivefold with sterile double‐distilled water. The PCR reaction mixture consisted of 5 µL SYBR Premix Ex Taq II (2×), 0.3 µL forward primer (10 µmol/L), 0.3 µL reverse primer (10 µmol/L), 1 µL cDNA template, and 3.4 µL sterile double‐distilled water. The PCR protocol included pre‐denaturation at 95°C for 30 s, followed by 40 cycles of 95°C for 5 s and 60°C for 34 s. The mRNA expression of the target gene was calculated using the 2^−∆∆CT^ method, −∆∆Ct = (Ct_target gene_ − Ct_β‐actin_)_treat_ − (Ct_target gene_ − Ct_β‐actin_)_control_ [49]. There were three repetitions per gene, and β‐actin was used as the housekeeping gene to quantitatively analyze the target gene.

### The analyses and calculations of apparent total tract digestibility

Feed and feces samples were oven‐dried at 105°C until constant weight, and all analyses were performed in duplicate. Gross energy (GE) was determined using a fully automatic precision calorimeter (MP‐C(X)2000) with benzoic acid as the calibration standard. Nitrogen content was measured according to the Kjeldahl method (GB/T 6432‐2018), and crude protein content was calculated by multiplying nitrogen content by the factor of 6.25. Acid‐insoluble ash (AIA) content was determined according to the incineration method described in GB/T 23742‐2009. The ATTD of nutrients was calculated as follows: Apparent total tract digestibility, % = 100 − [(AIA concentration in feed × nutrient concentration in feces)/(AIA concentration in feed in feces × nutrient concentration in feed)] × 100 [7].

### RNA sequencing

RNA was extracted from the jejunal mucosa of Shaziling and Yorkshire pigs and tested for integrity and purity. High‐quality samples were selected for constructing a sequencing library. Paired‐end RNA‐seq sequencing was performed using the Illumina HiSeq X Ten/NovaSeq. 6000 sequencer (2 × 150 bp read length) (Majorbio, China). To ensure data quality, raw reads containing more than 30% low‐quality bases (quality value ≤ 20 or N bases) were discarded, and only paired reads were retained to generate clean data. Clean reads were aligned to the reference genome in orientation mode using HISAT2 software. Differential expression analysis between the two pig varieties was conducted using DESeq. 2 software. Genes with |log2FoldChange | ≥ 1 and an adjusted *p*‐value (*Q* value) < 0.05 were considered significantly differentially expressed. KEGG pathway enrichment analysis results were visualized using bubble plots. GSEA was performed using the “clusterProfiler” package (version 3.14.3) in R (version 3.6.3).

### Gut untargeted metabolomics analysis and data processing

Intestinal samples from 210‐day‐old Shaziling pigs and Yorkshire pigs were weighed (50 mg each) and homogenized with 400 μL of extraction solution containing internal standards (methanol‐acetonitrile, 1:1 v/v; internal standard concentration: 2 μg/mL) using a high‐throughput tissue homogenizer under cryogenic conditions (60 Hz, −20°C). The homogenized samples were incubated at −20°C for 30 min, followed by vortex mixing (40 kHz) and ultrasonication. Afterward, the samples were maintained at 4°C for 30 min and centrifuged at 12,000 × *g* for 15 min at 4°C. The supernatant was collected for LC‐MS analysis, which was conducted at Shanghai Majorbio Bio‐Pharm Technology Co., Ltd. using an AB SCIEX UPLC‐TripleTOF system. Mass spectrometry data for intestinal metabolites were acquired in both positive and negative ion modes.

Raw data were processed for peak detection and alignment using Progenesis QI 2.3 software (Waters). Preprocessing steps included filtering low‐quality peaks, recoding missing values, data normalization, quality control (QC) sample validation (relative standard deviation, RSD < 30%), and data transformation. Principal component analysis (PCA) was performed using the ropls package (R packages, Majorbio). Differential metabolites were preliminarily screened via one‐way analysis of variance (ANOVA, *p* < 0.05) and further selected based on variable importance in projection (VIP > 1) from partial least squares‐discriminant analysis. Metabolic pathways enriched with differential metabolites were identified through the Kyoto Encyclopedia of Genes and Genomes (KEGG, http://www.genome.jp/kegg/) database (*p* < 0.05).

### Genotyping methods and results for PPARα knockout mice

According to the provided primer sequences (F1: 5'‐GACTTCACCGCATCTTTTAGACAC−3', R1: 5'‐GCAAGCTGAAATACACCAG GTG‐3', R2: 5'‐ACATGCGTGAACTCCGTAGTG‐3') and electrophoretic band identification results, the genotyping criteria for PPARα knockout mice are as follows: the homozygous mutant type shows a single band at 672 bp; the heterozygous type displays two bands at 672 and 873 bp; while the wild type exhibits a single band at 873 bp. There are 11 PPARα knockout homozygous mice in total, with identification numbers: 3, 4, 5, 9, 16, 18, 25, 36, 37, 50, and 51 (Figure S7).

### Intestinal organoids isolation and treatment

The jejunum was removed from the pig, opened longitudinally, and washed with ice‐cold PBS. Villi were gently scraped using a glass slide, and fat was removed as thoroughly as possible. Crypts were exposed by dicing the jejunum into 2 mm segments, followed by extensive washing to remove contaminants. The tissue fragments were transferred to a 50‐mL tube and washed repeatedly with pre‐cooled PBS until the supernatant was clear. Tissues were then incubated in PBS containing 2.5 mM ethylenediaminetetraacetic acid disodium salt (Sigma‐Aldrich) at 4°C on a rotator for 60 min to separate the epithelium. After removing the dissociation reagent, the tissue was vigorously mixed with PBS 25 times to obtain high‐purity crypts. The suspension was filtered through a 100 μm filter, and the crypts were resuspended in 10% (vol/vol) fetal bovine serum (FBS). The mixture was centrifuged at 1000 rpm for 5 min, the supernatant was discarded, and the pellet was resuspended in approximately 2 mL of medium. The resuspended crypts were then centrifuged at 600 × *g* for 5 min, and the supernatant was discarded. The crypts were suspended in Matrigel, and 40 μL of the Matrigel‐crypt mixture was added to the center of each well of a 24‐well plate. The plate was incubated at 37°C in a 5% CO₂ atmosphere for 20 min to allow the Matrigel to solidify. 500 μL of organoids culture medium was added to each well containing growth factors including Wnt3a, R‐Spondin 1, Noggin, N2 supplement (Gibco), B27 supplement (Gibco), n‐acetyl‐l‐cysteine (Sigma‐Aldrich), nicotinamide (Sigma‐Aldrich), epidermal growth factor (EGF, Sigma‐Aldrich), A83‐01 (TGF‐beta inhibitor, Tocris), SB202190 (p38 inhibitor, R & D Systems, Minneapolis, MN, USA), 10 μM Y27632 (Rho‐kinase inhibitor, R & D Systems), and 2.5 μM the glycogen synthase kinase 3 inhibitor (GSK3i, CHIR99021; Sigma‐Aldrich) and DMEMF12 medium (Gibco) containing 1% glutamine, penicillin/streptomycin, Hepes [50]. Intestinal organoids were cultured at 37°C in an incubator with 5% CO₂. The culture medium was completely replaced every 2–3 days.

After stable passaging, the organoids were incubated with DMSO, 60 µM WY14643 (S8029, Selleck), or 5 µM GW6471 (S2798, Selleck) [51]. To study the specificity of PPARα activation, organoids were treated with the endogenous PPARα ligand OEA (HY‐107542, MedChemExpress) at concentrations of 1 and 10 µM [52], or with palmitoylethanolamide (PEA; S4708, Selleck) at 10 µM [53]. To investigate the effects of NAEs on organoids, LEI‐401 (HY‐131181, MedChemExpress) was used [54], with NAEs containing 10 µM OEA and 10 µM PEA. In the OXPHOS model, organoids were treated with rotenone (S2348, Selleck) at a concentration of 0.05 μM [38]. The budding efficiency, budding budding length, and growth area of organoids were assessed and analyzed across different treatments. The growth area of organoids was quantified from images using Image‐Pro Plus 6.0 software. The area of the organoids was manually outlined using the “Freehand” selection tool and then measured in square pixels. This value was converted to square micrometers (µm²) based on the set scale. For each experimental condition, the growth areas of all organoids from at least three independent culture wells were measured and averaged. Intestinal organoids were harvested 3 days post‐treatment for RNA analysis.

For intestinal organoids in mice, the isolation procedure is identical to that used for pigs. However, the conditions for crypt digestion in mice differ: the tissue is incubated in a solution containing 2.5 mM ethylenediaminetetraacetic acid disodium salt (Sigma‐Aldrich) at 4°C on a rotator for 30 min. The crypts are then filtered through a 70 μm filter and resuspended in 10% (vol/vol) FBS. The culture medium for mice does not require Wnt3a but includes R‐Spondin 1, Noggin, N2 supplement, B27 supplement, n‐acetyl‐l‐cysteine, epidermal growth factor, and DMEM/F12 medium supplemented with 1% glutamine, penicillin/streptomycin, and Hepes.

### Dual‐luciferase reporter assay

The promoter region (−3000 to 0 bp) of the *ACAA2* gene was amplified by PCR and cloned into the XhoI/HindIII site of the pGL4.20 plasmid (Promega) to generate the pGL4.20‐ACAA2 dual‐luciferase vector. PCR Amplification of ACAA2. Primers: The *ACAA2* gene fragment was amplified using the following primer pair: Forward Primer (F): 5'‐CTGAGGCAGGAAAATCAC‐3'; Reverse Primer (R): 5'‐CTACTTCCTGGGGCTCCTT‐3'. Reaction Setup: The PCR was carried out in a total volume of 10 μL. The reaction mixture comprised: 5 μL of 2× Takara PrimerSTAR Max DNA Polymerase; 0.2 μL of each primer (10 μM stock concentration); 1 μL of human genomic cDNA template; 0.5 μL of DMSO; Nuclease‐free ddH₂O to a final volume of 10 μL. Thermal Cycling Protocol: Amplification was conducted under the following conditions: Initial Denaturation: 98°C for 3 min; Amplification Cycle (35 cycles): Denaturation: 98°C for 10 s; Annealing: 58°C for 15 s; Extension: 72°C for 90 s; Final Extension: 72°C for 10 min. Downstream Processing: The resulting PCR amplicons were purified using a gel extraction kit according to the manufacturer's instructions. The purified DNA fragments were then ready for ligation into a plasmid vector. The recombinant plasmid was confirmed by Prime Sequencing. 293T cells were cultured in DMEM supplemented with 10% FBS and 1% penicillin/streptomycin. For luciferase reporter gene assay, 293T cells were inoculated into 12‐well plates and co‐transfected with Lipofectamine 3000 transfection reagent (L3000‐015, Thermo Fisher Scientific, Waltham, MA) when the cell density was 60%–70% with pGL4.20‐ACAA2, pRL‐TK and either the PPARα overexpression vector (pcDNA 3.1‐PPARα) or the vector plasmid pcDNA 3.1. After 24 h of incubation, cells were treated with fresh medium containing DMSO or 5 µM OEA or 60 µM WY14643, with four replicates per group. After an additional 24 h, the cells were collected and lysed. Luciferase activity was measured using the Dual‐Luciferase Reporter Assay System (E1960, Promega). The relative firefly luciferase activity of the reporter plasmid was normalized by Renilla luciferase activity.

### Statistical analysis

During the data analysis stages, we indeed performed normality (Shapiro‐Wilk test) and homogeneity of variances (Levene's test) assessments for all datasets. All datasets met the assumptions required for two‐way ANOVA. At each developmental stage of Shaziling and Yorkshire pigs, the results of organ index, intestinal morphology, proliferation and differentiation of ISCs, brush border enzyme activity, and nutrient transporter mRNA expression were analyzed using ANOVA with the GLM procedure (SPSS, version 22.0; IBM Corp., Chicago, IL, USA) for the factorial arrangement of treatments, which included breed, age and their interaction. When a significant interaction or main effect was detected, post hoc multiple comparisons were conducted. Specifically, for detailed exploration of all pairwise differences following a significant interaction, Fisher's Least Significant Difference (LSD) test was applied. If a significant main effect was found, Duncan's new multiple range test was employed for multiple comparisons.

For comparisons between two groups, statistical significance was determined using an unpaired two‐tailed Student's *t*‐test. For comparisons among three or more independent groups under a single‐factor design, a one‐way ANOVA was performed, followed by Duncan's multiple range test for post hoc analysis if the overall *F*‐test was significant. The *p*‐values were calculated with a 95% confidence level. Data are presented as means ± SEM. Differences were considered significant when *p* < 0.05 and trending towards significance when 0.05 ≤ *p* ≤ 0.1. All graphs were plotted using GraphPad Prism 8.0 (GraphPad Inc.).

## AUTHOR CONTRIBUTIONS

**Qianqian Wang**: Conceptualization; methodology; software; data curation; writing—original draft; writing—review and editing; visualization; validation; funding acquisition. **Lanmei Yin**: Writing—review and editing; conceptualization; methodology; software. **Zhaobin Wang**: Software; data curation. **Jun Li**: Software; data curation; visualization. **Xianglin Zeng**: Validation; methodology. **Qiye Wang**: Investigation; resources. **Jianzhong Li**: Resources. **Kang Xu**: Methodology; visualization. **Huansheng Yang**: Funding acquisition; project administration; supervision; resources; writing—review and editing. **Yulong Yin**: Project administration; supervision; resources. All authors have read the final manuscript and approved it for publication.

## CONFLICT OF INTEREST STATEMENT

The authors declare no conflicts of interest.

## ETHICS STATEMENT

The ethics application (No. D2020039) was approved by the Research Ethics Committee of the University of Hunan Normal University, Changsha City, Hunan, China.

## Supporting information


**Figure S1.** The comparison of organ index and intestinal morphology between Shaziling and Yorkshire pigs at different ages.
**Figure S2.** The representative images of immunohistochemistry of Shaziling pigs and Yorkshire pigs.
**Figure S3.** The comparison of proliferation and differentiation of intestinal stem cells and nutrient absorption between Shaziling and Yorkshire pigs at different ages.
**Figure S4.** Schematic representation of the binding sites of PPARα with other genes.
**Figure S5.** PEA improves intestinal morphology via PPARα.
**Figure S6.** Oleoylethanolamine produced by lipid metabolism regulates intestinal stem cell activity and villus size via PPARα signaling pathway.
**Figure S7.** Genotyping methods and results for PPARα knockout mice.

**Table S1.** Ingredients and nutrient composition of OEA experimental piglet diet.
**Table S2.** Ingredients and nutrient composition of PEA experimental piglet diet.
**Table S3.** The primer sequences and lengths of the target genes used for jejunal mucosa and organoids.

## Data Availability

Raw sequencing data on intestines of Shaziling and Yorkshire pigs have been saved in the Sequence Read Archive (SRA) database Raw sequencing data on http://www.ncbi.nlm.nih.gov/bioproject/PRJNA1372273. Intestinal organoids of Shaziling and Yorkshire pigs have been saved in the Sequence Read Archive (SRA) database https://www.ncbi.nlm.nih.gov/bioproject/PRJNA1373145. Raw sequencing data on Intestinal organoids treated with OEA have been saved in the Sequence Read Archive (SRA) database https://www.ncbi.nlm.nih.gov/bioproject/PRJNA1372972. The data used are saved in GitHub https://github.com/286227/iMetaOmics. Supplementary materials (figures, tables, graphical abstract, slides, videos, Chinese translated version, and update materials) may be found in the online DOI or iMeta Science http://www.imeta.science/imetaomics/.
