# Supplementary material for: Oleoylethanolamide regulates intestinal stem cell activity and villus size via PPARα signaling pathway
Source: IMetaOmics. 2026 Jan 26;3(1):e70078. doi: 10.1002/imo2.70078 (PMC13674433; doi:10.1002/imo2.70078)
Supplement: Supplementary file 1 [file IMO2-3-e70078-s001.docx]

**Supporting information to** **Lipid Metabolism Drives Intestinal Morphogenesis: Oleoylethanolamide Regulates Intestinal Stem Cell Activity and Villus Size via PPARα Signaling Pathway**

**Running title**: Lipid Metabolism Drives Intestinal Morphogenesis

Qianqian Wang^1#^, Lanmei Yin ^1,2#^, Zhaobin Wang^3#^, Jun Li^1^, Xianglin Zeng^1^, Qiye Wang^1,2,4^, Jianzhong Li^1^, Kang Xu^2^, Huansheng Yang^1,2,4, *^, Yulong Yin^2,4^

^1^ Hunan Provincial Key Laboratory of Animal Intestinal Function and Regulation, Hunan International Joint Laboratory of Animal Intestinal Ecology and Health, Laboratory of Animal Nutrition and Human Health, College of Life Sciences, Hunan Normal University, Changsha, 410081, China.

^2^ Key Laboratory of Agro-ecological Processes in Subtropical Region, Institute of Subtropical Agriculture, Chinese Academy of Sciences; Hunan Provincial Engineering Research Center for Healthy Livestock and Poultry Production; Scientific Observing and Experimental Station of Animal Nutrition and Feed Science in South-Central, Ministry of Agriculture; Hunan Provincial Key Laboratory of Animal Nutritional Physiology and Metabolic Process, Changsha, 410125, China.

^3^ Department of Animal Science, University of Manitoba, Winnipeg, R3T 2N2, Canada

^4^ Yuelushan Laboratory, No. 246 Hongqi Road, Furong District, Changsha, 410128, China.

^#^ These authors contributed equally: Qianqian Wang, Lanmei Yin, Zhaobin Wang.

^*^ Correspondence: yhs@hunnu.edu.cn (Huansheng Yang).

**Figure S1 The comparison of organ index and intestinal morphology between Shaziling and Yorkshire pigs at different ages.** (A) Six each of 30, 60, 90, 150, 210, and 300 days of age Shaziling and Yorkshire pigs were selected to compare intestinal development. (B) Small intestine length. (C) Small intestine length index, the ratio of total small intestine length to body weight at euthanasia. (D) Small intestine weight. (E) Small intestine weight, the ratio of total small intestine weight to body weight at euthanasia. (F) Duodenum villus height. (G) Jejunum villus height. (H) Ileum villus height. (I) Duodenum crypt depth. (J) Jejunum crypt depth. (K) Ileum crypt depth. (L) Duodenum villus width. (M) Jejunum villus width. (N) Ileum villus width. (O) Duodenum VH:CD. (P) Jejunum VH:CD. (Q) Ileum VH:CD. (R) Villus surface area of duodenum. (S) Villus surface area of jejunum. (T) Villus surface area of ileum. VH:CD: villus height to crypt depth. At each developmental stage of Shaziling and Yorkshire pigs, the results of organ index and intestinal morphology were analyzed using analysis of variance (ANOVA) with the GLM procedure (SPSS, version 22.0; IBM Corp., Chicago, IL, USA) for the factorial arrangement of treatments, which included breed, age and their interaction. When a significant interaction or main effect was detected, post hoc multiple comparisons were conducted. Specifically, for detailed exploration of all pairwise differences following a significant interaction, Fisher’s Least Significant Difference (LSD) test was applied. If a significant main effect was found, Duncan’s new multiple range test was employed for multiple comparisons. Data are presented as means ± SEM (n = 6 biological replicates/group). Differences were considered significant when *p* < 0.05 and trending towards significance when 0.05 ≤ *p* ≤ 0.1. Significant differences between the two breeds at the same age are marked as^*^ for *p* < 0.05, ^**^ for *p* < 0.01, ^***^ for *p* < 0.001.

**Figure S2 The representative images of immunohistochemistry of Shaziling pigs and Yorkshire pigs.** (A) The representative images of Ki67-positive cells in crypts (magnification 40×; scale bar, 100 μm). (B) The representative images of endocrine cells in villi and crypts (magnification 40×; scale bar, 100 μm). (C) The representative images of goblet cells in villi and crypts (magnification 40×; scale bar, 100 μm).

**Figure S3** **The comparison of proliferation and differentiation of intestinal stem cells and nutrient absorption between Shaziling and Yorkshire pigs at different ages.** (A) The Ki67-positive cells number in jejunum crypt. (B-C) The endocrine cells number in jejunum villus and crypt. (D-E) The goblet cells number in jejunum villus and crypt. (F-H) Brush border enzyme activity between Shaziling and Yorkshire pigs at different ages. (F) Jejunum ALP. (G) Jejunum maltase. (H) Jejunum sucrase. (I-O) Relative mRNA expression of nutrient transporter carriers between Shaziling and Yorkshire pigs at different ages. (I) *SGLT1* (J) *GLUT2* (K) *SLC6A19* (L) *SLC7A1* (M) *SLC1A1* (N) *PepT1* (O) *SLC27A4*. (P) The comparison of apparent total tract digestibility of whole-gut gross energy between Shaziling and Yorkshire pigs at 210 days of age. (Q) The comparison of whole-gut apparent total tract digestibility of crude protein between Shaziling and Yorkshire pigs at 210 days of age. ALP: alkaline phosphatase. *SGLT1*: sodium glucose cotransporter 1, *GLUT2*: recombinant glucose transporter 2, *SLC6A19*: solute carrier family 6 member 19, *SLC7A1*: solute carrier family 7 member 1, *SLC1A1*: solute carrier family 1 member 1, *PepT1*: peptide transporter 1, *SLC27A4*: solute carrier family 27 member 4. At each developmental stage of Shaziling and Yorkshire pigs, the results of proliferation and differentiation of intestinal stem cells, brush border enzyme activity, and nutrient transporter mRNA expression were analyzed using analysis of variance (ANOVA) with the GLM procedure (SPSS, version 22.0; IBM Corp., Chicago, IL, USA) for the factorial arrangement of treatments, which included breed, age and their interaction. When a significant interaction or main effect was detected, post hoc multiple comparisons were conducted. Specifically, for detailed exploration of all pairwise differences following a significant interaction, Fisher’s Least Significant Difference (LSD) test was applied. If a significant main effect was found, Duncan’s new multiple range test was employed for multiple comparisons. Data are presented as means ± SEM (n = 6 biological replicates/group). Differences were considered significant when *p* < 0.05 and trending towards significance when 0.05 ≤ *p* ≤ 0.1. Significant differences between the two breeds at the same age are marked as^*^ for *p* < 0.05, ^**^ for *p* < 0.01, ^***^ for *p* < 0.001.

**Figure S4** **Schematic representation of the binding sites of PPARα with other genes.** (A, B) indicates binding sites for human sapiens PPARα and other genes and (C) indicates binding sites for Mus musculus PPARα and other genes. The PPRE sequence is from JASPAR ([JASPAR - A database of transcription factor binding profiles (elixir.no)](https://jaspar.elixir.no/). The binding sites of PPARα and the promoter sequences of human-derived *ACAA2*, porcine-derived *ACAA2*, and murine-derived *ACAA2* are highlighted in yellow below. *ACAA2*: acetyl-CoA acyltransferase.

**Human sapiens ACAA2 gene:putative PPARA binding site.**

>NC_000018.10:c49816533-49813485 Homo sapiens chromosome 18, GRCh38.p14 Primary Assembly

GATAATCGGGAGGCTGAGGCAGGAAAATCACTTGAACCTGGGAGGTGGAGGTTGCAGTAAGTGGAGATCA

TGCCACTGCACTCCAGCCTGGGCAACAAAGCAAGACTGTCTCAAAAAAAAAAAAAAGAAAAAGAATAATG

AACAACTGTATATTCTTCATTTGGATTCCCCAGTTGGTAATATTTGGCCATATTTGCTTTATCTTTCTCT

GCCAGTGTATAGTCATGCACTATCTTTCCTATGTTTAGATGCACAAATGCTTACTACTTGCTACAACTGC

CTACAGTGTTCAGTAGAGTAAAATGCTATACAGGTTTGTAGCCTAGGAGCAATAGGCTGTACCATATAGC

CTAGGTGTGTAGTAGGCTCTACCATTGAGGTTTGTGTAAGTGAAATCTATGATGTTCACACAATGACGAA

ATCGCCTAATAGCACATCGTTCAGGATGGATTCTCGTGGTTAAGCAACACATAACTGTATATATATAAAT

GCATTTTTTCCAGTCATCTGCAAGCTCATGATTCTTCACCCCCATATATTTCAGTATGTATTTCCTTAGA

ACAAAGGACAATCTTCTACATAACTACAATATAATTATCATGATCAGGAAACTTAATGTCAATATTACCA

TGTCATCTATAATTTATATGTTTCTCCAGTTGTCCCAATACTGTCTTTTAGAGTATTTTTTTAACCCAGG

TTTTAATTAAGGATCTCATACAATATGTGTCACATATCTAGTCTCCTTTAATGTGATACTATTCCTATTT

TAGCCATTTTTTGTTTTTCACCACACTGATGTTTTTAAAGAGTTCAGGTCAGTTGTTCTGTGGAATGTAC

CTCCATGTGGATTTGTCTCATTGTTTCCTTATAATTAGATTCAGGCCAGATGTTTTCAGCAGAAATTCTA

CGCAAGTGAGATTGTGTTGTCGCTGTAGCAAGGGGAGCGCATGACGTCAGTCTGTCCCTTTATTGGTGAT

GGGACACCATTACTTCATAATTTGGTGAAGTAATACCCGCAAGATTTATCCATTGTAAAGGTACCCTTTC

CTCTTCATGAGACTTGGAGACTATGTCAATATTCTCTCATATATTTACGTGTTTTAATATATGTAAACCT

TTCACTGAATGGTGTTAGAATCCACTCATTAGTGACATTTCCAATTAGAGAAAACACAGTACATCTATAA

AGACCTACTGATGGTGATCTGCAGTGGGAAGTTGTGATACTTAACCTTTCTTAAATATGTAGAGGCAAAC

TGAATAGAAAACTGTGTACAAAAATGAACACTCTATACATGAGCATTTTTTTTTTTTTTTTTTTTGAGAC

GGAGTCTTGCTCTGTCACCCAGGCTGGAGTGCAGTGGCACGATCTCGGCTCACTGCAAGCTCCGCCTCCC

AGGTTCACACCATTCTCCTGCCTCAGCCTCCCCAGCAGCTGGGACTACAGGCGCCCGCCACCACGCCCAG

CTAATTTTTTTGTATTTTTAGTAGAGATGAGGTTTCACCGTGTTAGCCAGGATGGTCTCGATCTCCTGAC

CTCGTGATCCGCCCACCTCGGCCTCCCAAAGTGCTGGGATTACAGGCGTGAGCCACGGCGCCCGGCCTAT

ACATGAGCATTTGTGAAGCTTGTCTAAGATTTGTACATTTGATTGCGTGGATATTTTGCCTTAAAAAGAA

CTGTAAATGAATAATGTGTGCTGATGTGTTCATGGGTGTAGAGTACTAAGGTCTGCATTAAAAAGATAAG

ATGGTTTGATGGAAGAACAGGTGGATAGCAATATAGCAAAATGTTAACAATTGGGTCGATATGTGTCCAC

TGTACAAGTCTTCCCACTTTTCTGTAAGTTTGTAAGTTTTCATAATACAAATTTGGATTTAAAAACACCC

CAACATTGCAAGAGTGTCACCAAAGTGTTTGAAAAGCAGGATTCTAGGGGGGGCATTCGCTGACGACTCA

GAGAAACCGTCCATGCCACCCACCACCCCTACGCACTCAAAAGAAGCCCTCGCTAGGCAGCCCGGCCCTC

CGAGCCCTGGTATCGAGGGGCTCTCCGCCTCTCCCCTGCCGCGGCCGCCTCCTGCTTGCCCGGCCTCAGT

CTGTTCTCAGAACATACTCCATCACCTGGTTCCCAGAACTCAGATTGCGCAGTGGTCTCGTCATCATCGG

CCAGGACTCACAGTGCCCGCGGCAGAGGCCTCCCTAGACCTCCCTCCCGTCCAGCCTCACCCGCTGCCTA

CTCTCCTCACGCCCCTGCTCCAGGTCCCCTGGCCCCATTTCGCTCCGTTTTCATAATCCTCTCAGG

CTCCGGGCAAGCGGCGCCGCCCGCAATGGGACCTGATCATATAAGGAAAATACTGCGGGCTCATCCGGGG

GCTGCAATGGGGACCCGAAAGCGCCCTAGCCTACTACAATCACCGCACCCCAACTGCCGCCCACCTCTCT

GCCCTGCCCCCAGTACAGTACCCAAAACCGGTCCGGGTCCAGCTCGGCGGCCCAAGCCTCTGCCCAGCGG

ACTGCGCACCCGCCCGCCCCAGCCAATCCCGCCGCCGCCGCCGCGCCCCGCCCACAGGACGTTCGGTCCC

GCCCCCAGCTGGCGGCCGCGGCCGCCCGCGCGCCAAGTTCCTCAGCCCTTGGCTCCTGCCCAGTGTTTAG

GGTGTTGGCGGAGACAAAGGGGAAGAGTCATCGCCTGTCGGGGCTAGGATATGATGGGTGAGAGGTGTCA

AACCAAATTCTCTCGGTTTGGAAACGGAGAAAATCTAAAAATGAGGATGTGAGGAAAGAGTCCGCTCTCA

AGGCGCGTTGTGGTCTATCCGAGCCCCGTCCCCTGGGCTCCCTCGGGCTGGGGTGAGGCGGGCAGCGCTA

CGCGTGGGGTAGGACCATCTTACGCTGGGACCCCGCCAAGGAGCCCCAGGAAGTAGGTGAAAGGGCAGGG

GCGTGGCTCTCGGGGCGCCACCCACGCTCTTGAAATCTGGGTGATTGCGAGCGGCCGCTCAGCGTCCCCC

ACACCACAGACCCGCGCCGCCGACGACCCAGCAGCCGCC

**Sus scrofa ACAA2 gene:putative PPARA binding site.**

>NC_010443.5:c99313759-99310759 Sus scrofa isolate TJ Tabasco breed Duroc chromosome 1, Sscrofa11.1, whole genome shotgun sequence

GATATTATCATACTAATGAGGTGAAACAGAGAGAGAAAAATATGACATCACTTATAAATGGAATCTAAAA

TATGACACAGGAGCTCCTGCTGTGGTACAGTTTAAGATTAGCAGATGCAAACCATTATATATGCAAACCT

ATATATATATAGGATGGATAAACAACAAGCCCCTACTTGTTGGGACTACACTCAAGAGCCCCACTCAATA

TCTTATAATAAACTATAATGGGAACTATATTCAATATCTTGTAATAAACTATAATGGAAAAGAATGTGAA

AAATAATATATATTTTTAATTAAATCACTTTGCTATGCAGTAGAAATTAACACAACATTGTAAATCAACT

CTACCTCAATAAAATATTTTTTAGAAAAACTAAAATGTTGAGTTCCTGTTGTGGTTCACTGGGTTAAGAT

CCCAATATCCATGAGGATGCAGGTTCAATCCCTGGCCTTGCTTAGTGGGTTAAGGATCTGGTGTTGCTGT

GAGCTGTGGTGCAAATCACAGATGTGCCTTAGATCCCATGTTGCTGTGGCTGTGGCATAGGCTGGCAGCT

GCAGTTCAGATATAACCCTGAGGTGGGAACTTCTAGATGCCACAGGTGCAGGCCTAAGAAGCAAAAAATA

AATAAATAAAATAAAATGTTGTCATAGTTCAAAAAAAAAAAAAAAAAAAGAGAGAGAGAGAAAGGAAAAA

CCCCTACCTATTTCATGGAGTTGCAAAATGTCCCTCAATTTGGATTTGTCTGATGTTATGTGAATTATGT

TTTTCTGTTTTGTACAATTCTCATATTGCCTGAGTGTAAAAATTATAAACGGCGTTCATAAGTGCTTGTT

TCAATGCTGGACAAACAGTTCCCAAATGTGGCAGTTTTATTTTACTATCACTCTCTGCTCATCCTCTAGA

ATATGCCGTGGGTGTGACCCTAAAAAGACAGAAAGACAAAAATAAATAAATAAATAAATAAATACAATGT

TAATAATCTTAGAATTTCTGTATTGAATCGATGACTGTCAATTTCCTGGATTTGCTCAAATGAAAGAAAC

AGAAAAAGAAAAGGAATAAACCAGAGGATCGAATTACAATAGCTGGTGAGAAAAAGGGAACAATGGCTAA

ATGGGAGTTGGATGGGAAATGAATGATGTACATCTGTAACTATGGACATGTTCTAATTACATTTTGAAAG

CCTTGAGTGGTTTGGATTACAAGAATCCAATTCAATCCTGAACACATGGAAATTTTCCCTTAAAAGAGTA

AACAAATACTGACCTCTAACATGAACTAAGGTATTCCTGGGGTGAAGTGTACTAAGGTCTGCGTAAATAA

ATAAATAAGATGGTTTGATGGAAGAAGAGAGGAAATGATACAGCAAATATGGCAAAATGTTAAGGGTAGA

ATACAGTCAGTGAATATACCAGTGTTTGCTCTTTCTATGTTTCTTTCTACTTTTCTGTAAGTTCGAGAAA

GTTTTCACAATAACAACACTGGGTTAAAAAAAATAGGAGTTCTTGCTGCAGCGCCGGGATGCAGTTTTGA

TACCCCACCCAACACGGAGCCAGTGGTGTAAAACGCTAGAGCAGCTCTGATCTGATCCCTGACCCAGGAA

CTTCCATATGCCGAGTGGCGGCCCAAACAAAAAAAAAAACAAAAACAAAAACAAAACACCCCAAACCCAA

ACAAACCAAAAAAAAAAAAAAAAAACCCACAACAACAAACGATGTGACAGGGCCACGAAAGTGTTTGAAG

AGAAGGCATTCGCTGTTGGCTCAGAAAACCTCAGAGCCTCCCACCACCCCTTATCAGAAGCCTGAGGCAG

CCTGGCCGGCCTCCAAGCCCTGGCGCTGAGAATCTGCCCCATCTCTGCCCGGATCGCCTCCTGCTACCCT

GGTCTCAGATCGTTCTCAGCACAAACTCCATCACCTGGTTCCCAGATCTCAGATTGCGCAGTGGTCACGT

CATCATCGACCAGGGCTCACAGTTACAGCGGCAGAGGCATCCCTAACCTCCCTCATCCCTAGCCTAACCA

GCTGCCCCTCCTCTTCCCCCCAACCCCCGTTCCCGGCCCCCACCTCGCCAGTCACGTATTATAATCCTCT

CAGGCTCCAGGCCAGCAGCAGCGCTGCCAAGCCATGGGGCCCAATCATATAAGGAAAATACTGCGGCTTC

ATTCGGGGCCTGCAAAGTGGAACAGGGGGCGCCTCCGCCCTAAAACAGTCGCTACACTCCTAGGGCGCAA

ACCTCCCCGCCCCCTCCCGGGACCTATTTCCCCTCCCCCGCCCAGAGGCCTAGGCCTTTGCCAGCGGTCT

GGGCAGTGGCCTACCCAACCTGCAAAATGGGCTTAGCCAATCCCCCCTCGCCATCTTCCACGCTCCGCCC

ATTCGACTTCCGGCAGCGCCGCACCAACTGATCTTCAGCTTTTGCGGAAGGTGGGGCAGGTTTATAAAGG

CTGGGAAGAGAATGGATAGGGTAGAGTCAAATTATTTTTTTTTTCCCCCTGGGGGAATAGAGAATGGGAG

TTTCCGTTGAGGAAATGAACCTGACTAGTGCCCATGAAGATAAGGTTCATTCCTGGCCTCGCACACTCAG

TGGGTTAAGGATCTATAGATAGCAAATGGGGCTAGGATCTAGTGTTGCTGTGACTGTGGATAGGCCGGCA

GCTGCAACTCGGATTGGACCCCCTAGCCCGGGAACTTCCGTATGACGTGGCTGTGGCCCTAAAAAAAAAA

AAAAAAAACCACACAGAGATAAAGTCCTAAAAACGATGTGAGGGAAAAATCCGCTCACGAGGACCCTAGT

CCCAGTGCCTCCGCCCCCACCCAGTCCTGTGGGATGGCGCTTAGAGGGCTGAGAGTTCCAAAGCGGCGGA

TAACTGGCTGGGTGCCCAATGTGTGAGAAGGCAGGGGCGCGGCCCTGCTGGCACCGCCCCCTCGCTCCTA

AAGTCTGCAGGACCTCGCGCGGCCGGTCTGCATCGAGGAGGACCCTACGCTGCAGCCACCA

**Mus musculus ACAA2 gene:putative PPARA binding site.**

>NC_000084.7:74909283-74912283 Mus musculus strain C57BL/6J chromosome 18, GRCm39

AGAAGAAAAGAGCTCTCAAGTCAAGGCCAAGACAATGCCTCACAGCTACCAGAATTCACTAAAACAAAGT

AAACTATAACAAGCAAGCTCAGCCGGGCGTGGTGGCCACGTCTTTAATCCCAGCACTCGGGAGGCAGAGG

CAGGAGGATTTCTGAGTTTGAGGCCAACCTGGTCTACAGAGTGAGTTCCAGGACAGCCAGAGCTACACAG

AGAAACCCTGTCTCGAAAAACCAAAAAACAAGCAAGTTCTTTATCCTTGTTTGGCAACTTGAACTTCCTG

GAGAACAGATGTTTGTAAAACAAACAAACAAACAAACCACAGCAGCCAAGCTACTATTTGCCTTCTACCT

GAAGTTCTCACCAACCCAGACTGTGCACATGTGCACTGATGCAGGGTCTTCTTATCTAGTCAATGCTGAC

TCGAACTTAGCATCTTCTTCACCCTCTCTCTCAAGTGCTATGACTATAGGCATGAGCCGTAGTCTCAGCA

TTGTCCACTTGCTCTAAAACTGTTGCGGCTCCCAGGATGTCACCTGCTCTCCTCAGCTACCAGTACTGTA

ACTTCTAGCAATGCTGAATATTACAGGAAGAGGGGGGAAACTGTGTTAACTATGGGGACATAGCCCAGAA

AGTGTGGAAGAAAGGACCGAAGCTGGACCATTCTGAGACCCTGAAAAGCTCTTAAACAGTAGAATTTACA

GATTTACAATTGACTCATTTCCAGTTTTATATGAACACATCTTCCCCTCTAATATTAACTACTGAAAGAA

ATTAACACTGCATTTCTAAAAACACTGGAGGCTAACCCTGCTAAGAACAGAACAGTAAGACTAGCTCAGA

AGTCCCATGCCTAGGCTCCTAAATTGTATTCTGAAAGGCACAGAATTATTTACCTACTTATTAGGTGTAC

CTACCTAAATAGCGACTTAATTAGAGATTACCTTAAGTAACTTTGTAGCTTAGTTGAAGGAAACTTTGCC

CCCCCCCCCGTGTGTGTGGGGGGGGGGGGGGGTGAAGAATGTCAATCTCCAGAAGAAAAGGGGAAACAGA

ACAGAGCTTAGGACACTTTCCCATTCTTCAGTAACAGTTAGTTTTGCCACGTTTTCCTTCTTGCTACTGT

TTCTATCCATTTTTGTCCTGCATGACTTTAGTTGCAGATACTGAATCTTCACCCTTAAACATTTCTGTGT

ACAACTACAAACAAACCAAAAAACCTCCAAAAGCCAGTCCCCACATGCCAATTAATCTTGGGCTTGTCTC

AGCATGATTAAACTCAGGGCACTTAGAGGAGGAATCCTACATAAGCGCGTTAAGTCCTCCGTGCTCAAGG

GAGTCCACGGCCTCCTTACTGGTGACTCTAAGTCTGGTCATTTGGTTAAGGAAAAGCCTCCACTCCTTTT

CTGAGACTGAAACTGCAGATATTCTGACACATACCTGTTTGATTTCACACTGATTTTTCAGGGCCCGCTC

ACATAATTACAGTCAACAGTAAGCACGGAGTCCCTCTTGGTAGTGACTTGAAGGGCATCCGAAACAGGGA

ACCCATGCAAGAGCAAAGACAGATATCTGTTCCTCTAGCTTGAACTTTGCAGTCGCTGCGTGAAAATTCC

TAAGAGAACTGTAACAAACGTGTGTGTGTGTGTGTGTGTGAAGTGTACATAGGGTGAAGTAAACGTGCAA

GGTTTTAAAATACAGTTTTAATGAGTAGATGTGAAACAGCAAACAGTATGTTCCCCAACACGAACCTTGC

CACGTGTCTGACGTTTTCACAAAAAATAAAAAATAAATAAATAAATAATTTTTTTTTAAAAAGGTGGTTA

GACCCAGGTGTGGTGAAGCACGCCTTTAACTCCAGCATTTAGGCGAATCACATAATTCCTAGCTTAGTCT

ATAGGGCGAGTTTCAGAACAGCAGATAACCCTGTTAGGAGAAAGAAAAAAGAAAAAAAAAGCGGGAGAGA

GAGATTAAAAAGGTAGTCCCCACTTCAAGGGAGCTGGTGAAACACACACAAAGCTGGCTTAAGGGTAGAC

AGTGATTGACGAGTTAAAAGAAAGCAACAGCACCACCCACGCCCTGTTCCCACGCTCAGCCCAGGCCACT

TGGCCGCCCCGGGGCCCCGAGCTCTTATCTTCCCCTCGCCCCGGGCCCCTCTCTCTGCTTAGGCCGCCTC

CAGCTCGCCTGGCCTCAGTGTTCTCAGCACACTCCATCACCTGGTTCCCAGAACTCAGATTGCGCAGTGG

TCATGTCATCATTGGCAGGGCTTGCAGCTGGAGGCGTGGGTACCCCTAAACCTATCCCCTCCCCGAAGCC

TGGCCTCCCCACTCAGTCCCTCTCCCCTCTGCTTCCCCGCTCCAGGTCCCCTGACCCCATCACCTGCCAC

GTTTTCATAATCCTCTCAGGCTCCAAACCAGCAGCGCCGGTTACGAGAGGCCTGATCATATAAGGAAAAT

ACATCGGGCTCACTCGGGGCTGCCTCGGGGACCCAAGCGCTCCCTGCTCTACCACGTTCCCCTTCCCTTC

TCTAGACGCCCCAGCTACCCGCCACCGCCCCGCTCCGACCTAGCACAGGCCGCCGCCAGAAGGCTGCGCA

GGCGCAAGGGCAGCCAATCCCACCGCACCGCCGCCAGCCCCGCCCCCTGATAACAGGTCCCGCCCTCCAC

CAGTCGGCCCCGCCCGTTCCCCATCCCCCCACACCCCACCTCCGCTTTTTCTGGGATGTGGGGTGGGGTT

GATGCTGAGTTCTTGGCTCCAGTCCTGTCCTGGAGAAAGCGAGTGGGCGGTGCCTCTAGAAGCCTGGGAT

AGGGCAGATGGGGTGTGCGGTCGAAGTGGTTCTCAAATTCCCAGAGGAGGGGCGCGGGGCACACGTGGCC

CGAAGTGGGGGTAACCTTGGTCTTCCCGAGGCTCGGGGGCGAGGATGGGGAAGTAGGGGAAAGGGCAGGG

GCGCGGCCTCTCGGGGCTCCGCCCACACTCTTAAGGCTGGGCTAGGGCTGGTGGCCGGGTG

**Figure S5 PEA improves intestinal morphology via PPARα.** (A) The budding efficiency, the budding crypt depth, crypts per organoid and the growth area of of primary organoids in control and PEA group. (n = 6 biological replicates/group). (B) The representative images of primary organoids in control and PEA group (scale bar, 100 μm). (C) The budding efficiency, the budding crypt depth, crypts per organoid and the growth area of Second-generation organoids in control and PEA group. (n = 6 biological replicates/group). (D) The representative images of Second-generation organoids in control and PEA group (scale bar, 100 μm). (E) The villus height, crypt depth, villus width, VH:CD and villus surface area of the middle small intestine. (F) The mRNA relative expression of PPARα target gene and fatty acid oxidation gene in control and PEA group. (G) The mRNA relative expression of *PPARγ* and *PPARδ* gene in control and OEA group. (H) The mRNA relative expression of *PPARγ* and *PPARδ* gene in control and PEA group. VH:CD: villus height to crypt depth. *CPT1A*: Carnitine Palmitoyltransferase 1A, *PPARα*: Peroxisome proliferator-activated receptor alpha, *PDK4*: pyruvate dehydrogenase kinase 4, *ACOX1*: acyl-Coenzyme A oxidase 1, *PPARγ*: Peroxisome proliferator-activated receptor gamma, *PPARδ*: Peroxisome proliferator-activated receptor delta. For comparisons between two groups, statistical significance was determined using an unpaired two-tailed Student's t-test. Data are presented as means ± SEM (n = 8 biological replicates/group). Significant differences marked as^*^ for *p* < 0.05, ^**^ for *p* < 0.01, ^***^ for *p* < 0.001.

**Figure S6 Oleoylethanolamine produced by lipid metabolism regulates intestinal stem cell activity and villus size via PPARα signaling pathway.** This image was Created with BioRender.com. FFAs: Free fatty acids. OEA: Oleoylethanolamine. PPARα: peroxisome proliferator-activated receptor Alpha. RXR: Retinoid X Receptor. ‌PPRE: peroxisome proliferator responsive elements.

Figure S7 **Genotyping methods and results for PPARα knockout mice.**
